# Economic evaluation of telephone-based weight loss support for patients with knee osteoarthritis: a randomised controlled trial

**DOI:** 10.1186/s12889-018-6300-1

**Published:** 2018-12-27

**Authors:** Kate M. O’Brien, Johanna M. van Dongen, Amanda Williams, Steven J. Kamper, John Wiggers, Rebecca K. Hodder, Elizabeth Campbell, Emma K. Robson, Robin Haskins, Chris Rissel, Christopher M. Williams

**Affiliations:** 1 School of Medicine and Public Health, Hunter Medical Research Institute, University of Newcastle, Newcastle, NSW 2308 Australia; 2 Hunter New England Population Health, Locked Bag 10, Wallsend, NSW 2287 Australia; 3 Centre for Pain, Health and Lifestyle, Ourimbah, NSW Australia; 4 Department of Health Sciences, Faculty of Science, Vrije Universiteit Amsterdam, Public Health Research Institute, Amsterdam, Netherlands; 5 Department of Health Sciences, Faculty of Science, Vrije Universiteit Amsterdam, MOVE Research Institute, Amsterdam, Netherlands; 6 School of Public Health, University of Sydney, Lvl 10, King George V Building, Camperdown, NSW 2050 Australia; 7 Outpatient Services, John Hunter Hospital, Hunter New England Local Health District, Locked Bag 1, New Lambton, NSW 2305 Australia; 8 NSW Office of Preventive Health, Liverpool Hospital, South West Sydney Local Health District, Locked Bag 7279, Liverpool BC, NSW 1871 Australia

**Keywords:** Osteoarthritis, Knee, Cost-effectiveness, Obesity, Telephone, Weight loss

## Abstract

**Background:**

The prevalence of knee osteoarthritis is increasing worldwide. Obesity is an important modifiable risk factor for both the incidence and progression of knee osteoarthritis. Consequently, international guidelines recommend all patients with knee osteoarthritis who are overweight receive support to lose weight. However, few overweight patients with this condition receive care to support weight loss. Telephone-based interventions are one potential solution to provide scalable care to the many patients with knee osteoarthritis. The objective of this study is to evaluate, from a societal perspective, the cost-utility and cost-effectiveness of a telephone-based weight management and healthy lifestyle service for patients with knee osteoarthritis, who are overweight or obese.

**Methods:**

An economic evaluation was undertaken alongside a pragmatic randomised controlled trial. Between May 19 and June 30, 2015, 120 patients with knee osteoarthritis were randomly assigned to an intervention or usual care control group in a 1:1 ratio. Participants in the intervention group received a referral to an existing non-disease specific 6-month telephone-based weight management and healthy lifestyle service. Quality-adjusted life years (QALYs) was the utility measure and knee pain intensity, disability, weight, and body mass index (BMI) were the clinical measures of effect. Costs included intervention costs, healthcare utilisation costs (healthcare services and medication use) and absenteeism costs due to knee pain. Data was collected at baseline, 6 weeks and 26 weeks. The primary cost-effectiveness analysis was performed from the societal perspective.

**Results:**

Mean cost differences between groups (intervention minus control) were $493 (95%CI: -3513 to 5363) for healthcare costs, $-32 (95%CI: -73 to 13) for medication costs, and $125 (95%CI: -151 to 486) for absenteeism costs. The total mean difference in societal costs was $1197 (95%CI: -2887 to 6106). For QALYs and all clinical measures of effect, the probability of the intervention being cost-effective compared with usual care was less than 0.36 at all willingness-to-pay values.

**Conclusions:**

From a societal perspective, telephone-based weight loss support, provided using an existing non-disease specific 6-month weight management and healthy lifestyle service was not cost-effective in comparison with usual care for overweight and obese patients with knee osteoarthritis.

**Trial registration number:**

ACTRN12615000490572, registered 18th May 2015

**Electronic supplementary material:**

The online version of this article (10.1186/s12889-018-6300-1) contains supplementary material, which is available to authorized users.

## Background

Osteoarthritis is one of the fastest growing chronic health problems worldwide [1, 2]. According to the 2015 Global Burden of Disease Study, osteoarthritis accounted for 3.9% of years lived with disability worldwide in 2015, up from 2.5% in 2010, and was the 13th highest contributor to global disability [1, 2]. Knee osteoarthritis consistently accounts for approximately 85% of the burden attributable to osteoarthritis [1, 2]. Osteoarthritis also imposes a significant economic burden, with the total annual costs estimated to be $A8.6 billion in Australia [3], £20.9 billion in the UK [4], and $US142.1 billion in the US [5]. The majority of these costs are attributable to ambulatory and inpatient care, including surgery and lost work productivity [3, 4].

Excess weight is an important modifiable risk factor for the onset and progression of knee osteoarthritis [6], and there is strong evidence that weight loss interventions reduce pain and disability in overweight patients with knee osteoarthritis [7, 8]. Consequently, international clinical practice guidelines recommend all patients with knee osteoarthritis who are overweight receive support to lose weight [9–11]. Typically, these treatments are delivered using clinical face-to-face models of care [12]. While such clinical models produce moderate effects on weight loss, pain, and physical function [7, 8], only 22% of patients with knee osteoarthritis referred for orthopaedic consultation at a large Australian public hospital report receiving weight loss care [13], possibly due to limitations in service delivery and patient access to care. Arguably more scalable delivery options, using remotely delivered approaches, such as telephone-based support, can maximise the reach of weight loss care and are more cost-effective to support weight loss in this patient group. While telephone-based behavioural interventions targeting weight loss are used routinely in the general populations, the cost-effectiveness of referring patients with knee osteoarthritis to these is unknown.

Given the scarce resources in healthcare, policy-makers are increasingly requiring evidence of economic value for healthcare interventions to make informed decisions about how to allocate resources [14]. Therefore, undertaking economic evaluations of knee osteoarthritis management approaches is important. Recently, we conducted a randomised controlled trial (RCT) using an existing non-disease specific telephone-based weight management and healthy lifestyle service for patients with knee osteoarthritis who are overweight or obese [15]. The primary objective of the intervention was to reduce knee pain intensity, by reducing weight. The RCT found no between-group differences in knee pain intensity, nor weight [15]. Conducting a cost-effectiveness analysis is recommended in all trials, irrespective of their clinical effect [14]. This recommendation is based on cost-effectiveness analyses considering the joint distribution of differences in cost and clinical effect and thereby is able to show that an intervention is cost-effective when neither cost nor clinical effect differences are individually significant [14]. Cost-effectiveness analyses estimate the cost (saved or spent) per unit of effect gained. Such estimates can support healthcare policy and decision-makers choose which interventions should be implemented for specific health outcomes given the available resources [14]. The purpose of this study is to undertake an economic evaluation of the aforementioned RCT, compared to usual care.

## Methods

### Study participants and design

The economic evaluation was conducted alongside a pragmatic parallel group RCT, which was part of cohort multiple RCT [16]. Full details of the study design has been described in the paper presenting the clinical results of the trial [15] and in the study protocol [17, 18]. The trial was prospectively registered (ACTRN12615000490572). The Hunter New England Health Human Research Ethics Committee (13/12/11/5.18) and the University of Newcastle Human Research Ethics Committee (H-2015-0043) approved the RCT.

Patients on a waiting list for an outpatient orthopaedic consultation for their knee osteoarthritis at the John Hunter Hospital in NSW, Australia, were invited to participate. Patients were assessed for eligibility during a telephone assessment and eligible patients were randomised into either the intervention or usual care control group (1:1 ratio).

Inclusion criteria were: primary complaint of pain due to knee osteoarthritis lasting longer than 3 months; 18 years or older; overweight or obese (body mass index (BMI) ≥27 kg/m^2^ and < 40 kg/m^2^); average knee pain intensity ≥3 out of 10 on a 0–10 numeric rating scale (NRS) over the past week, or moderate level of interference in activities of daily living (adaptation of item 8 of SF36); and access to a telephone. Exclusion criteria were: known or suspected serious pathology as the underlying cause of their knee pain (e.g. fracture; cancer, inflammatory arthritis; gout; or infection); previous obesity surgery; currently participating in any prescribed or commercial weight loss program; knee surgery in the last 6 months or planned surgery in the next 6 months; unable to comply with the study protocol that requires them to adapt meals or exercise due to non-independent living arrangements; medical or physical impairment precluding safe participation in exercise such as uncontrolled hypertension; and unable to speak or read English sufficiently to complete study procedures. Recruitment for the trial occurred from May 19 to June 30, 2015, and follow-up concluded January 26, 2016.

### Interventions

The intervention included two components. First, brief advice and education about the benefits of weight loss and physical activity for knee osteoarthritis were provided over the telephone immediately after randomisation. Second, intervention participants were informed about the NSW Get Healthy Information and Coaching Service (GHS) (www.gethealthynsw.com.au) [19], and referred to the service for weight loss support. The GHS is an existing government funded telephone-based health coaching service developed to support adults of the general population to make sustained healthy lifestyle improvements. Targets include diet, physical activity and achieving a healthy weight, and if suitable, referral to smoking cessation services [19]. The GHS provides 10 individually tailored coaching calls, centered on national dietary and physical activity guidelines [20, 21], delivered over a 6-month period by university qualified health professionals [19]. Participation in the intervention did not affect the patients’ place on the waiting list for orthopaedic consultation.

Participants in the control group remained on the ‘usual care pathway’ (i.e. on the waiting list to have an orthopaedic consultation and could progress to consultation if scheduled or surgery if recommended by the orthopaedic department) and took part in data collection during the 6-month intervention period. No other active intervention was provided as part of the study, however; no restrictions were placed upon the use of other health services. Control participants were informed that a face-to-face clinical appointment was available in 6 months with a study physiotherapist (CW).

### Measures

All measures were collected by self-reported questionnaires at baseline, six weeks and 26 weeks (see Additional file 1: Appendix 1 for questionnaires). The baseline questionnaire was completed by telephone. Week 6 and week 26 surveys were completed via telephone or mailed in the post as per participant preference.

### Utility measure

We measured utility using health-related quality of life, assessed using the 12-item Short Form Health Survey version 2 [22]. Participants’ SF-6D [23] health states were converted into utility scores using the British tariff [24]. QALYs were calculated by multiplying the duration of time spent in a health state by the participants’ utility score and were linearly interpolated between measurement points.

### Clinical measures of effect

#### Primary outcome

Knee pain intensity was assessed using an 11-point NRS. Participants were asked to rate their “average knee pain intensity over the past week”, where 0 represents ‘no pain’ and 10 represents ‘the worst possible pain’ [25].

#### Secondary outcomes

Disability was assessed using the Western Ontario and McMaster Universities Osteoarthritis Index (WOMAC) [26]. The total WOMAC score ranges from 0 to 96, with higher scores indicating greater disability. Weight (kg) was assessed via participant self-report and BMI was calculated as weight/height squared (kg/m^2^) [27] using self-reported weight and height.

### Cost measures

Costs were converted to Australian dollars in 2016 using the consumer price index [28]. As the follow-up of the trial was 26 weeks, discounting of costs was not necessary [29].

Intervention costs were estimated using a micro-costing approach and included the cost of delivering the telephone brief advice at baseline and the cost of the GHS coaching calls. The cost to deliver the brief advice was calculated by estimating the development and operational costs of the call and the estimated wages for the telephone interviewer to provide the brief advice (estimated average time 5 min). The cost to provide the GHS coaching calls was provided by the GHS [30] and multiplied by the number of calls each participant received. The GHS reported the number of health coaching calls participants received directly to the research team.

Healthcare utilisation costs were calculated from a patient reported healthcare utilisation inventory and included any healthcare services or medications used for knee pain (independent of the intervention costs). Participants were asked to recall the type of healthcare provided and the number of sessions attended as well as all medications used for their knee pain during the past six weeks, within the six and 26 weeks follow-up participant surveys. Healthcare services were priced according to Australian standard costs or professional organisations if this data was unavailable [31–33]. Medications were priced using unit costs from the Australian pharmaceutical benefits scheme [34] or online Australian pharmacy websites if this data was unavailable. To gain an estimate of the cost of healthcare utilisation over the entire 26-week period, the average of the week six and week 26 costs per patient was interpolated, assuming linearity.

Absenteeism was measured by participant recall of the total number of sickness absence days from paid work due to knee pain during the past six weeks, within the six and 26 weeks follow-up participant surveys. The ‘Human Capital Approach’ [29] was used to estimate absenteeism costs which involved multiplying each participant’s total number of sickness absence days off by the Australian Bureau of Statistic’s reported age and gender specific national average hourly income [27, 34]. To gain an estimate of the cost of absenteeism over the entire 26-week period, the average of the week six and week 26 costs per patient was interpolated, assuming linearity.

### Statistical analysis

Data were analysed in STATA (V13, Stata Corp). The sample size was based on the primary clinical measure of effect [15]. Analyses were performed according to the intention-to-treat principle. Descriptive statistics were used to compare baseline characteristics between the intervention and control group participants. Missing data for all effect and cost measures were imputed using Multiple Imputation by Chained Equations [35]. The imputation model included variables related to the “missingness” of data and those that predicted outcome variables, imputations were stratified by treatment group. Variables in the model included baseline education level, employment status, Aboriginal and/or Torres Strait Islander status, age, country of origin, gender, and duration of knee pain. Ten different datasets were created (loss-of-efficiency < 5%) [35]. These separate datasets were analysed as indicated below, after which pooled estimates were calculated using Rubin’s rules [36].

Mean cost differences between study groups were calculated for total and disaggregated costs. The cost measures were adjusted for the confounders of baseline knee pain intensity, baseline duration of knee pain, baseline BMI and number of days on the waiting list for orthopaedic consultation because the addition of these confounders to the regression model changed the cost differences by more than 10%. Total cost (ΔC) and effect (ΔE) differences were estimated using seemingly unrelated regression analyses, adjusted for baseline values as well as other potential baseline prognostic factors (knee pain intensity, duration of knee pain, BMI and number of days on the waiting list for orthopaedic consultation, obtained from hospital records) [37]. Seemingly unrelated regression is advantageous because possible correlation between the two regression equations (i.e., one for ΔC and one ΔE) can be accounted for [37].

Incremental cost-effectiveness ratios (ICERs) were calculated by dividing the adjusted difference in total costs between both groups by the difference in effects (i.e. ΔC/ΔE). Bias-corrected and accelerated bootstrapping (5000 replications) was used to estimate 95%CIs around cost differences and the uncertainty surrounding the ICERs. Uncertainty surrounding the ICERs was illustrated graphically using cost-effectiveness planes [29]. Cost-effectiveness acceptability curves, which consider the joint uncertainty of costs and effects, were used to graphically represent the intervention’s probability of cost-effectiveness in comparison with usual care at different values of willingness-to-pay [29].

The primary analysis was conducted from the societal perspective, which included all of the cost categories.

### Sensitivity analysis

We performed a per-protocol sensitivity analysis from the societal perspective that included only participants that completed at least six telephone GHS coaching calls in the intervention group (*n* = 20 participants).

### Secondary analysis: Healthcare perspective

A secondary analysis was performed from the healthcare perspective, which excluded absenteeism costs.

## Results

**Fig. 1:**
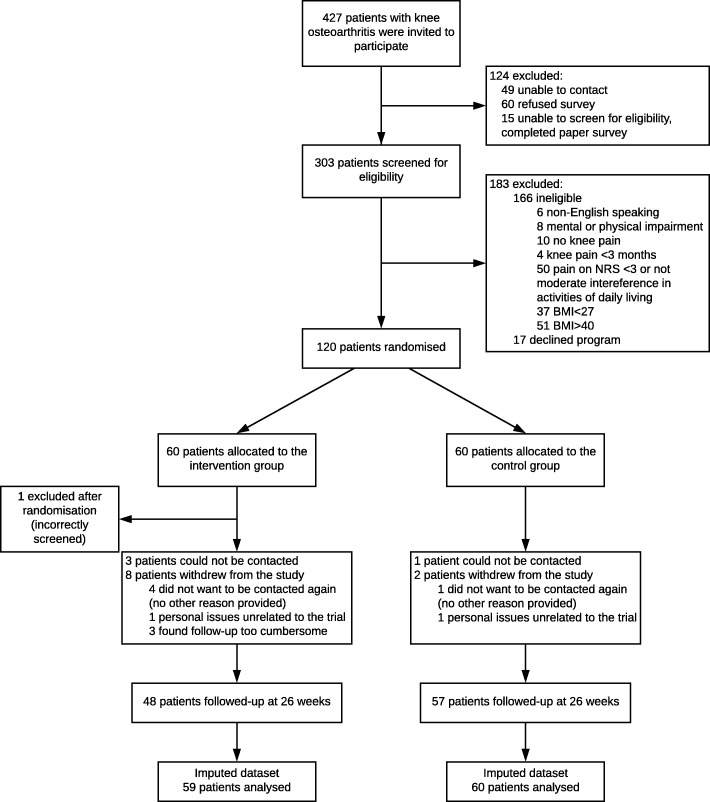
Flow diagram of trial participants

A total of 120 patients were randomised into the study (Fig. 1). Baseline participant characteristics were similar between groups (Table 1). Eleven participants in the intervention group and three in the control group were lost to follow-up. At 26 weeks, complete effect data was obtained from between 70 and 82% of participants (QALYs 70%, knee pain intensity 82%, disability 79%, weight 81%, BMI 81%). For cost data, complete data was obtained from 48% of participants at 26 weeks. As a consequence, between 18 and 30% of effect data and 52% of cost data were imputed.

**Table 1:** Baseline characteristics of the study population^a^

Demographic characteristics	Intervention group (*n* = 59)	Control group (*n* = 60)
Age (years)	63.0 (11.1)	60.2 (13.9)
Gender (male), n (%)	20 (34)	25 (42)
Aboriginal and/or Torres Strait Islander, n (%)	5 (9)	2 (3)
Employment status, n (%)		
Employed	12 (20)	14 (23)
Unemployed	7 (12)	8 (13)
Retired	31 (53)	28 (47)
Can’t work (health reasons)	9 (15)	10 (17)
Country of origin (Australia), n (%)	54 (92)	51 (85)
Highest level of education (>High school), n (%)	11 (19)	17 (28)
Private health insurance, n (%)	1 (2)	5 (8)
Current time on the waiting list for orthopaedic consultation (days), median (IQR)	379.0 (279.0–507.0)	390.0 (313.0–532.0)
Clinical characteristics		
Pain intensity (NRS)	6.9 (1.8)	6.8 (2.0)
Pain duration (years)	9.6 (10.6)	6.7 (8.5)
Disability (WOMAC)	47.9 (17.4)	48.6 (16.5)
Self-reported weight	93.3 (12.9)	89.5 (13.5)
Subjective BMI	33.4 (3.4)	32.1 (3.1)
Utility score	0.6 (0.1)	0.7 (0.1)
Healthcare utilisation, n (%)^b^	47 (80)	50 (83)

*IQR* Interquartile range

^a^Data presented as mean (SD) unless otherwise indicated

^b^Healthcare utilisation includes healthcare visits and medication use for knee pain

### Utility and clinical measures of effect

There were no differences found between groups for QALYs (Mean difference 0.00, 95%CI: -0.02 to 0.02), knee pain intensity (Mean difference 0.64, 95%CI: -0.49 to 1.77), disability (Mean difference 0.80, 95%CI: -6.68 to 8.47), weight (Mean difference − 0.02, 95%CI: -3.46 to 3.42), and BMI (Mean difference 0.11, 95%CI: -1.16 to 1.39) (Table 2).

**Table 2:** Differences in pooled mean costs and effects (95% CI), incremental cost-effectiveness ratios, and the distribution of incremental cost-effect pairs around the quadrants of the cost-effectiveness planes

Analysis		Outcomes	∆C (95% CI)	∆E (95% CI)	ICER	Distribution CE-plane (%)			
			AUD	Points	AUD/point	NE^c^	SE^d^	SW^e^	NW^f^
Primary analysis^a^	Societal perspective	QALYs	1197 (−2962 to 6139)	0.00 (−0.02 to 0.02)	581,828	26.2	15.5	20.8	37.5
		Pain intensity	1197 (− 2945 to 6126)	0.64 (−0.49 to 1.77)	1858	6.2	5.8	30.3	57.6
		Disability	1197 (− 2884 to 6151)	0.80 (−6.86 to 8.47)	1495	21.7	19.4	17.0	41.9
		Weight	1197 (− 2941 to 6153)	-0.02 (−3.46 to 3.42)	−58,194	30.6	18.8	17.6	32.9
		BMI	1197 (− 2864 to 6122)	0.11 (−1.16 to 1.39)	10,455	26.8	16.1	20.3	36.8
Sensitivity analysis^b^	Per protocol	QALYs	− 958 (− 5801 to 2790)	0.00 (−0.03 to 0.04)	−203,221	24.3	36.8	24.5	14.4
		Pain intensity	−958 (− 5803 to 2869)	0.70 (−0.75 to 2.15)	− 1370	6.3	10.9	50.6	32.2
		Disability	−958 (− 5819 to 2792)	1.21 (− 9.43 to 11.85)	−790	17.8	26.2	35.3	20.6
		Weight	−958 (− 5782 to 2804)	1.04 (−4.48 to 6.55)	− 922	13.1	22.8	38.6	25.5
		BMI	−958 (− 5785 to 2884)	0.62 (−1.42 to 2.65)	− 1553	10.3	18.4	43.1	28.1
Secondary analysis^a^	Healthcare perspective	QALYs	798 (− 3175 to 5686)	−0.00 (−0.02 to 0.02)	−387,820	24.1	17.5	24.0	34.3
		Pain intensity	798 (− 3197 to 5835)	0.64 (−0.49 to 1.78)	1238	5.9	6.3	35.3	52.5
		Disability	798 (− 3203 to 5663)	0.80 (−6.9 to 8.47)	994	19.7	21.4	19.7	39.2
		Weight	798 (− 3234 to 5670)	−0.21 (− 3.46 to 3.42)	−38,598	28.4	21.4	19.8	30.3
		BMI	798 (− 3281 to 5618)	0.11 (−1.16 to 1.39)	6968	24.9	18.3	23.1	33.7

*C* Costs, *E* Effects

Note: costs are expressed in 2016 Australian Dollars (AUD)

^a^Intervention n = 59, Control n = 60

^b^Intervention n = 20, Control n = 60

^c^The northeast (NE) quadrant of the CE plane, indicating that the intervention is more effective and more costly than control

^d^The southeast (SE) quadrant of the CE plane, indicating that the intervention is more effective and less costly than control

^e^The southwest (SW) quadrant of the CE plane, indicating that the intervention is less effective and less costly than control

^f^The northwest (NW) quadrant of the CE plane, indicating that the intervention is less effective and more costly than control

### Costs

The average number of GHS coaching calls to intervention participants was 4.7 (Standard deviation 4.6). The mean intervention costs were $622 (Standard error 80) per participant (Table 3). An overview of the unit costs and sources is reported in Table 4.

**Table 3:** Mean costs per participant in the intervention and control groups, and unadjusted and adjusted mean cost differences between study groups during the 6-month follow-up period (based on the imputed dataset)

Cost category	Intervention *n* = 59 mean (SE)	Control *n* = 60 mean (SE)	Unadjusted mean cost difference CI (95%)	Adjusted mean cost difference^a^ CI (95%)
Intervention	622 (80)	0 (0)	622 (474 to 788)	609 (461 to 796)
Healthcare	3346 (2453)	3487 (2001)	140 (− 4071 to 3952)	493 (− 3513 to 5363)
Medication	107 (21)	139 (28)	−32 (−73 to 7)	− 32 (−73 to 13)
Absenteeism	310 (157)	193 (93)	118 (− 123 to 424)	125 (− 151 to 486)
Total	4387 (2471)	3819 (2011)	568 (− 3436 to 4685)	1197 (−2887 to 6106)

Note: costs are expressed in 2016 Australian Dollars

Negative difference values indicate control group costs greater than intervention

^a^Mean cost difference (intervention minus control) adjusted for the baseline variables: knee pain intensity, duration of knee pain (years), body mass index, number of days on the waiting list for orthopaedic consultation

**Table 4:** Unit costs used for valuing resource use

Cost type	Unit of measure	Unit cost ($)^a,b^
*Intervention costs per participant* (1)		
*Healthcare services* ^c^		
General practitioner (3)	Consult	37.05
Medical specialist (4)	Consult	401.92
Chiropractor (2)	Consult	76.6–90.4
Physiotherapy (2)	Consult	76.6–90.4
Dietitian (2)	Consult	76.6–90.4
Other allied health (2)(3)	Consult	76.6–175.64
Massage therapy (2)	Consult	58.75–72.9
Alternative medicine (2)	Consult	75–128.75
Emergency (4)	Visit	456.05–714
Hospital admission (4)	Admit	4422.31
Spinal injection (3)	Injection	62.50–466.67
Imaging (3)	Test	177.45–179.20
Community services (2)	Consult	47.36–287
Orthopaedic surgeon consultation (4)	Consult	238.39
Pain clinic (3)	Consult	153.15
*Medications* (5)(6)		
*Absenteeism costs* (7)		

Sources of unit costs: (1) Bottom-up micro-costed; (2) Australian Medical Association; (3) Medicare Benefits Scheme; (4) Costs of Care Standards; (5) Australian pharmaceutical benefits scheme; (6) Online Australian pharmaceutical websites; (7) Average hourly income from the Australian Bureau of Statistics

^a^Costs are expressed in 2016 Australian Dollars (AUD)

^b^Some unit costs are reported in ranges due to difference in Initial versus follow-up consults and/or variation in healthcare services included under the same cost type

^c^Emergency refers to participants who presented to emergency department but were not admitted. Other allied health professional includes Back Fit. Alternative medicine refers to acupuncture. Community services refer to Novocare (homecare and transport) and home care

From the societal perspective, mean cost differences between groups (intervention minus control) were 493 (95%CI: -3513 to 5363) for healthcare costs, $-32 (95%CI: -73 to 13) for medication costs, and $125 (95%CI: -151 to 486) for absenteeism costs. The total mean difference in societal costs was $1197 (95%CI: -2887 to 6106) (Table 3). From the healthcare perspective, total mean difference between groups (intervention minus control) was $-1071 (95%CI: -5910 to 2931).

### Societal perspective: Cost-utility

**Fig. 2:**
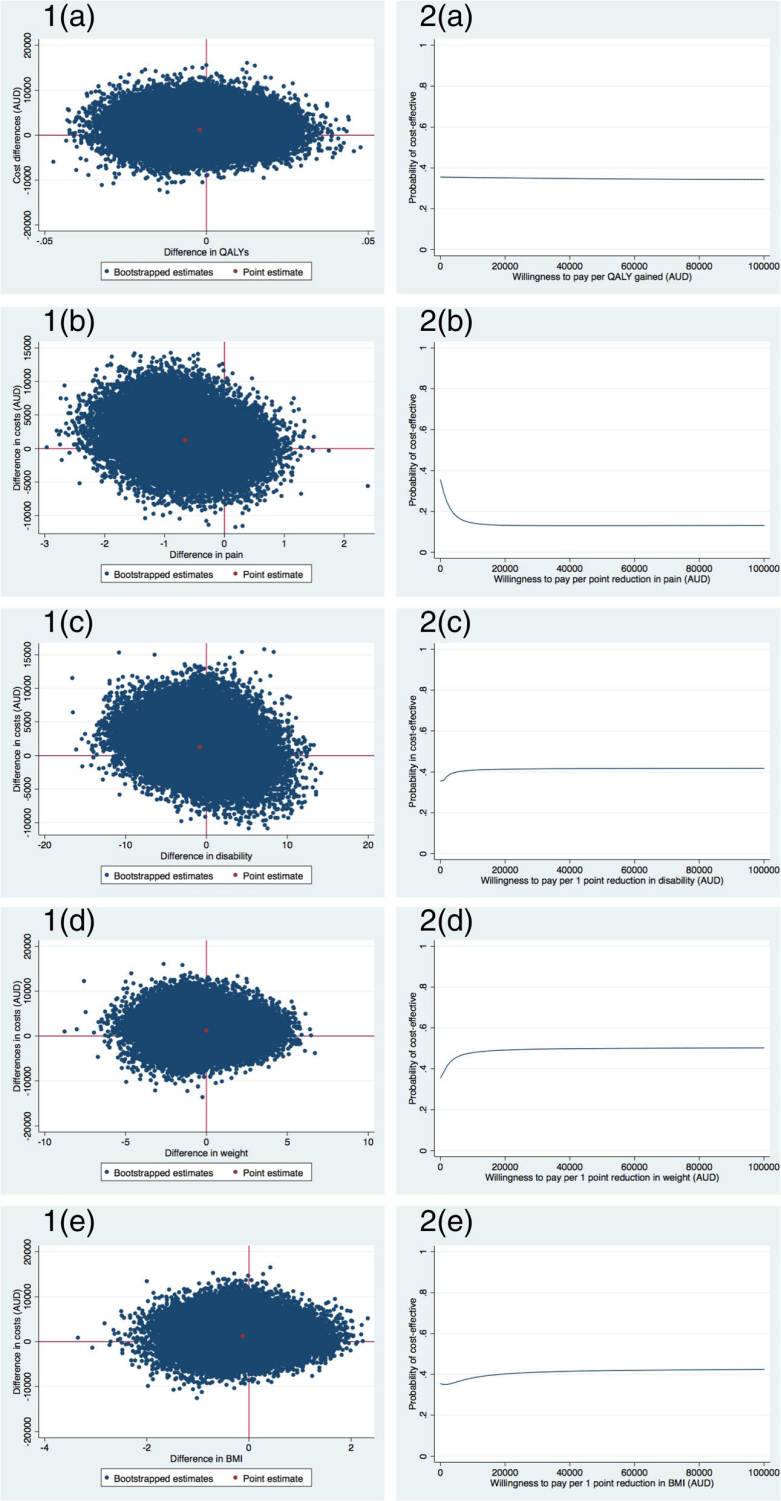
Primary analysis (societal perspective): Cost-effectiveness planes indicating the uncertainty around the incremental cost-effectiveness ratios (Fig. 2 (1)) and cost-effectiveness acceptability curves indicating the probability of cost-effectiveness for different values ($) of willingness-to-pay per unit of effect gained from the societal perspective (Fig. 2 (2)) for QALY (**a**), pain intensity (**b**), disability (**c**), weight (**d**), and BMI (**e**) (based on the imputed dataset)

For QALYs, most of the incremental cost effect-pairs were located in the northwest quadrant (37.5%), indicating the intervention was on average more costly and less effective than usual care (Fig. 2 (1a)). The ICER for QALYs was 581,828 indicating that one QALY gained in the intervention group was associated with a societal cost of $581,828 as compared with the control group (Table 2). This large ICER is due to the large difference in cost (Mean difference 1197 (95%CI -2962 to 6139) and very small effect on QALYs (Mean difference 0.00, 95%CI: -0.02 to 0.02). The cost-effectiveness acceptability curve for QALYs in Fig. 2 (2a) indicates the probability of the intervention being cost-effective in comparison to usual care was 0.36 at a willingness-to-pay of $0/unit of effect gained and the probability remained about the same irrespective of the willingness-to-pay.

### Societal perspective: Cost-effectiveness

For all clinical measures of effect, most of the incremental cost effectiveness-pairs were located in the northwest quadrant (Table 2, Fig. 2 (1b-1e)), indicating that the intervention on average achieved poorer outcomes at a higher cost compared to usual practice. Figure 2 (2b-2e) presents the cost-effectiveness acceptability curves for knee pain intensity, disability, weight, and BMI.

For clinical measures of effect, the probability of the intervention being cost-effective in comparison to usual care was 0.35 at a willingness-to-pay of $0/unit of effect gained. For disability, weight, and BMI the probability remained about the same irrespective of the willingness-to-pay (Fig. 2 (2c-2e)). For knee pain intensity, this probability decreased with increasing values of willingness-to-pay (Fig. 2 (2b)).

### Societal perspective: Sensitivity analysis

**Fig. 3:**
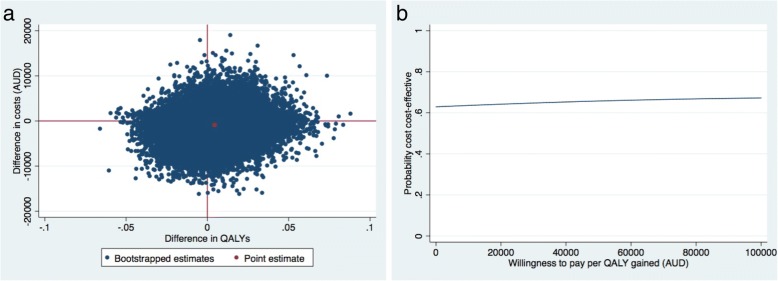
Sensitivity analysis: Cost-effectiveness plane indicating the uncertainty around the incremental cost-effectiveness ratios (**a**) and cost-effectiveness acceptability curves (**b**) indicating the probability of cost-effectiveness for different values ($) of willingness-to-pay per unit of effect gained for QALY

Results of the sensitivity analysis can be found in Table 2. In brief, for QALYs, the probability of cost-effectiveness was 0.63 at a willingness-to-pay of $0 per QALY gained (Fig. 3 (b)). For QALYs the probability of cost-effectiveness remained about the same irrespective of the willingness-to-pay.

### Healthcare perspective: Cost-utility

**Fig. 4:**
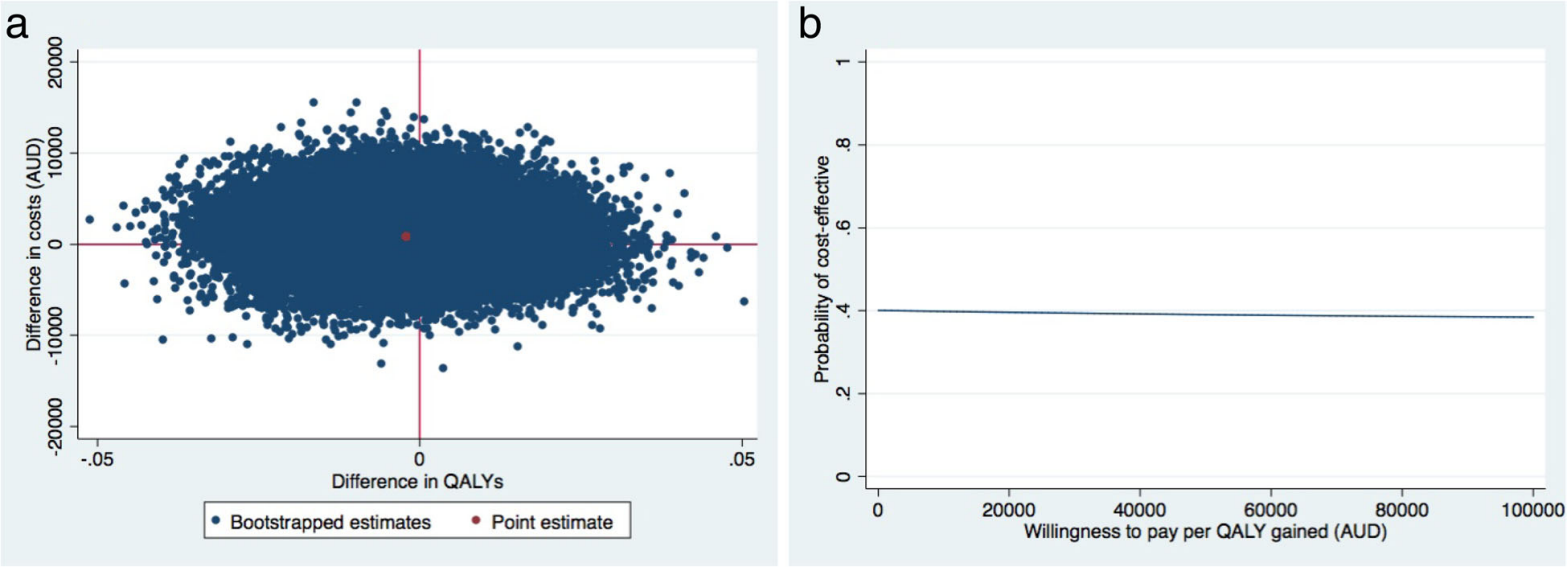
Secondary analysis (healthcare perspective): Cost-effectiveness planes indicating the uncertainty around the incremental cost-effectiveness ratios (**a**) and cost-effectiveness acceptability curves (**b**) indicating the probability of cost-effectiveness for different values ($) of willingness-to-pay per unit of effect gained from the healthcare perspective for QALY

The ICER for QALY was 387,820 indicating that one QALY gained was associated with a cost of $387,820 compared with the control group (Table 2). The probability of the intervention being cost-effective in comparison to usual care was 0.40 at a willingness-to-pay of $0/unit of effect gained remained about the same irrespective of the willingness-to-pay (Fig. 4 (b)).

## Discussion

We found that referral to a telephone-based weight management and healthy lifestyle service was not cost-effective from a societal perspective for patients with knee osteoarthritis who are overweight or obese, compared with usual care. The maximum probability of the intervention being cost-effective was low (≤0.40) for all outcomes for both the societal and healthcare perspectives, irrespective of the willingness-to-pay. The findings from the sensitivity analysis found the intervention had a slightly higher probability of cost-effectiveness compared to the main analysis, however the probability was still relatively low (i.e. 0.63 at a willingness-to-pay of $0/QALY) and remained the same regardless of willingness-to-pay.

To our knowledge, there are no other economic evaluations of telephone-based interventions for patients with knee osteoarthritis, hampering comparisons to similar interventions. A recent study assessed the cost-effectiveness of a 6-week multidisciplinary face-to-face treatment program compared with a telephone-based program for patients with osteoarthritis [38]. In this study, all patients received in-depth education about osteoarthritis, pain management, physical activity and diet, with the overall goal to enhance self-management skills [38]. Patients in the face-to-face group received six therapeutic groups session (2–4 h each), whereas the telephone group received only two face-to-face group sessions (2–2.5 h each) and four individual telephone contacts (15–30 min each). The study found that from a societal perspective the face-to-face treatment was more likely to be cost-effective when QALYs were the measure of benefit at 1-year follow-up [38]. Together with the findings from our current study, these results suggest that telephone-based care for patients with osteoarthritis may not be a cost-effective management approach. Since many patients with osteoarthritis do not receive recommended treatments via clinical models of care [13, 39], understanding why telephone-based interventions are reported to be as effective as face-to-face interventions but not cost-effective, is an important consideration to inform how best to provide care to this patient group.

An important strength of the present study is the pragmatic trial design, which enabled us to evaluate the intervention under ‘real world’ circumstances. This facilitates the generalisability of the results and allows decision-makers to use these results to help guide future healthcare interventions. A second strength is the use of contemporary statistical analysis methods. Multiple imputation was used to avoid loss of power due to sample size reduction and inefficiency associated with complete-case analyses and is regarded as a more valid way to deal with missing data than naïve imputation techniques, such as mean imputation or last observation carried forward [40]. Seemingly unrelated regression analyses were used for analysing the cost and effect components of the cost-effectiveness analysis. This method was used, instead of two separate regression analyses (i.e. one for costs and one for effects) or a net benefit framework, as it allowed us to adjust for various potential confounders that may not be the same for costs and effects, while simultaneously accounting for the possible correlations between costs and effects [37]. Bootstrapping techniques were used allowing for an estimation of the mean difference in costs as well as the joint uncertainty of costs and effects, while dealing with the right skewed nature of cost data.

This study is not without limitations. Firstly, the sample size calculation was based on the primary clinical measure of effect. While there have been a range of techniques proposed to estimate sample size based on economic endpoints [41–43], sample size calculations are usually performed based on the primary outcomes of the study [14, 44]. This is because cost data are right skewed and so require larger sample sizes than needed for trial outcomes to detect significant differences, which may not be feasible [14, 45]. Moreover, when performing a sample size calculation for economic endpoints a number of parameters need to be specified in advance e.g. cost measures, variance parameters of effectiveness measures, and incremental cost-effectiveness ratios, many of which are difficult to predict a priori [41–43]. As a result, economic evaluations conducted alongside clinical trials are typically underpowered [44] and should be interpreted with caution [45]. However, as economic evaluations are about estimation rather than formal hypothesis testing they still provide valuable information even when underpowered [29]. A second limitation is the rate of missing data at 26 weeks, between 18 and 30% for effect measures and 52% for cost data is high, however, not dissimilar to those in other economic evaluations [46]. Multiple imputation was used to account for the missing data, which is recommended over complete case analyses, still the results from this study should be treated with caution. Another limitation is that the self-reported cost data was interpolated to gain an estimate of costs over the 26-week intervention period as the recall period for participant’s healthcare utilisation and absenteeism was six weeks. Although it would be preferable for the recall period to cover the complete duration of follow-up we chose a shorter recall period to reduce participant recall bias. Lastly, presenteeism costs were not included, (i.e. reduced productivity while at work) which is known to be an issue reported by patients with chronic disabling pain [47].

There is a need for more information about the cost-effectiveness of lifestyle interventions for osteoarthritis. Although this study indicates that the use of a generic non-disease specific telephone-based service is not cost-effective for overweight and obese patients with knee osteoarthritis, the current evidence suggests existing models of care delivery are unable to provide recommended care to the large number of patients with knee osteoarthritis [13]. More research into how to provide scalable models of care that are cost-effective is needed. A potential way forward is to develop and test a range of scalable modes of weight loss care delivery e.g. telephone, online platforms (website, email), and mobile apps, and determine how these work together, or not, to deliver effective recommended care to patients. Importantly these various models of care delivery need to focus on delivering the same care e.g. focus on diet, exercise, to ensure that the studies are testing the mode of delivery, not the intervention components.

Interestingly, the National Institute for Health and Clinical Excellence guidelines for the management of osteoarthritis [9] refer to general obesity management guidelines for weight loss care for these patients [48], and not disease-specific models of care. Based on the results of our study, recommending general non-disease specific weight loss interventions may not be appropriate, or cost-effective for these patients. A key feature of our study and that of other osteoarthritis telephone interventions is that they only provide support over a relatively short period (six weeks to six months). However, other general weight loss programs occur over a much longer time frame. Better understanding about how the key ingredients for telephone services like dose and relevant components (e.g. exercise, weight loss, education) affect cost-effectiveness may provide more insight about the true value of telephone-based approaches for osteoarthritis.

## Conclusions

Our findings suggest that referral to a telephone-based weight management and healthy lifestyle service is not cost-effective compared with usual care for overweight and obese patients with knee osteoarthritis. These findings apply to QALYs, knee pain intensity, disability, weight, or BMI, from the societal and healthcare system perspectives.

## Additional file

Additional file 1: **Appendix 1.**Patient Questionnaire (Baseline) (DOCX 678 kb)

## Abbreviations

BMI: Body mass index
GHS: Get healthy information and coaching service
ICER: Incremental cost-effectiveness ratio
QALYs: Quality-adjusted life years
RCT: Randomised controlled trial
WOMAC: Western Ontario and McMaster Universities Osteoarthritis Index
