# Supplementary material for: Economic evaluation of telephone-based weight loss support for patients with knee osteoarthritis: a randomised controlled trial
Source: BMC Public Health. 2018 Dec 27;18:1408. doi: 10.1186/s12889-018-6300-1 (PMC6307168; doi:10.1186/s12889-018-6300-1)
Supplement: Supplementary file 1 — Appendix 1.Patient Questionnaire (Baseline) (DOCX 678 kb) [file 12889_2018_6300_MOESM1_ESM.docx]

## Appendix 1: Patient Questionnaire (Baseline)

**Contact Details**

**First Name:** _____________________ **Family Name:** _______________________

**Address:** ___________________________________________________________

___________________________________________________________________

**Primary contact number:** _____________________________________________

**Mobile number:** _____________________________________________________

**Email address:** _____________________________________________________

**Demographics**

1. **What is your date of birth? ____/____/______**
2. **Are you of Aboriginal or Torres Strait Islander Origin?**

 Yes, Aboriginal Origin

 Yes, Torres Strait Islander Origin

 Yes, both Aboriginal and Torres Strait Islander Origin

 No

1. **What is your current employment status?**

 Employed full time

 Employed part-time/casual

 Unemployed

 Can't work - health reasons

 Home duties

 Student

 Retired

 Other (please specify):___________________

1. **In which country were you born?**

 Australia

 Other country (please specify):______________________________________

1. **What is the highest level of education you have achieved?**

 Never attended school

 Some primary school

 Completed primary school

 Some high school

 Completed School certificate / Intermediate / Year 10 / 4th Form

 Completed HSC / Year 12 / 6th Form

 TAFE certificate or diploma

 University or College degree or higher

1. **Apart from Medicare, are you covered by private health insurance?**

 None

 Private hospital only

 Private ancillary only

 Private hospital and ancillary

 DVA (Department of Veterans’ Affairs)

1. **In the last two months have you had any medical condition for which you needed to take medication or receive medical attention? Tick all that apply.**

 Heart disease, e.g. heart attack, angina, Ischaemic heart disease, heart failure

 Stroke

 High blood pressure

 High blood cholesterol

 Blood clot (or thrombosis)

 Diabetes

 Osteoporosis

 Cancer

 Asthma

 A lung or respiratory condition, e.g. bronchitis or emphysema, but NOT asthma

 Multiple Sclerosis

 Neurological disorder, such as Parkinson’s disease

 Back pain

 Depression/Anxiety/Stress

 Other significant illness (please specify): ________

1. **Are you currently taking any medications for your knee pain?**

🞎 Yes – please specify below 🞎 No – continue to the next question

Please specify each medication name and duration (in weeks, months and/or years e.g. 2 years 6 months)

| **Medication name** | **Duration (length of time you have been taking this medication)** |
| --- | --- |
|  |  |
|  |  |
|  |  |
|  |  |
|  |  |

**Health service utilisation for your knee pain**

Did you use any health services in the **last 6 weeks** for your knee pain? This includes seeing a general practitioner (GP), a physiotherapist or dietitian, any hospital admissions or use of any community health or other services?

🞎 Yes – please specify below 🞎 No – continue to the next question

| **Type of service** | **Number of sessions** | **Estimated out of pocket cost** |
| --- | --- | --- |
|  |  |  |
|  |  |  |
|  |  |  |
|  |  |  |
|  |  |  |

**Weight and Height**

1. **Do you know how much you currently weigh?** If you do not know please provide an estimate.

________ Kilograms / stones / pounds (please circle unit of measurement)

1. **How tall are you without shoes on?** If you do not know please provide an estimate.

________ Centimetres / feet / inches (please circle unit of measurement)

**Pain Characteristics**

1. **How would you your average knee pain over the LAST WEEK on a scale from 0 to 10, where 0 is no pain and 10 is the worst possible pain. Please circle a number below.**

This is before you take any type of medication to help with your pain.

| **0** | **1** | **2** | **3** | **4** | **5** | **6** | **7** | **8** | **9** | **10** |
| --- | --- | --- | --- | --- | --- | --- | --- | --- | --- | --- |

No pain Worst possible pain

1. **How long have you been troubled with your knee pain?** Please enter a number in both years and months e.g. if 2 months would be entered as: 0 years and 2 months

Years: *_______________________________*

Months: *_____________________________*

1. **Do you REGULARLY experience pain in other bodily areas?**

 Yes – please tick all bodily areas that apply to you below

 No – continue to the next question

 Head

 Neck

 Shoulder

 Spine

 Hand

 Wrist

 Elbow

 Arm

 Hip

 Back

 Foot

 Heel

 Ankle

 Other:___________

1. **How many days off your normal PAID WORK did you have due to your knee pain in the last 6 weeks?** If you are not employed or did not have any days off please write 0

______________________Days

1. **During the PAST WEEK, how would you rate your sleep quality overall?**

| 🞎 Very good | 🞎 Fairly good | 🞎 Fairly bad | 🞎 Very bad |
| --- | --- | --- | --- |

**WOMAC**

1. The following questions concern the amount of pain you are currently experiencing in your knees. For each situation, please enter the amount of pain you have experienced in the **past 48 hours**

|  | None | Mild | Moderate | Severe | Extreme |
| --- | --- | --- | --- | --- | --- |
| 1. Walking on a flat surface | 🞎 | 🞎 | 🞎 | 🞎 | 🞎 |
| 1. Going up or down stairs | 🞎 | 🞎 | 🞎 | 🞎 | 🞎 |
| 1. At night while in bed | 🞎 | 🞎 | 🞎 | 🞎 | 🞎 |
| 1. Sitting or lying | 🞎 | 🞎 | 🞎 | 🞎 | 🞎 |
| 1. Standing upright | 🞎 | 🞎 | 🞎 | 🞎 | 🞎 |

1. Please describe the level of pain you have experienced in the **past 48 hours** for each one of your knees

|  | None | Mild | Moderate | Severe | Extreme |
| --- | --- | --- | --- | --- | --- |
| Right knee | 🞎 | 🞎 | 🞎 | 🞎 | 🞎 |
| Left knee | 🞎 | 🞎 | 🞎 | 🞎 | 🞎 |

1. How severe is your stiffness after first awakening in the morning?

| None | Mild | Moderate | Severe | Extreme |
| --- | --- | --- | --- | --- |
| 🞎 | 🞎 | 🞎 | 🞎 | 🞎 |

1. How severe is your stiffness after sitting, lying, or resting later in the day?

| None | Mild | Moderate | Severe | Extreme |
| --- | --- | --- | --- | --- |
| 🞎 | 🞎 | 🞎 | 🞎 | 🞎 |

1. The following questions concern **your physical function**. By this we mean your ability to move around and to look after yourself. For each of the following activities, please indicate the **degree of difficulty** you have experienced in the **last 48 hours**, in your knees.

What degree of difficulty do you have with:

|  | None | Mild | Moderate | Severe | Extreme |
| --- | --- | --- | --- | --- | --- |
| 1. Going down stairs | 🞎 | 🞎 | 🞎 | 🞎 | 🞎 |
| 1. Going up stairs | 🞎 | 🞎 | 🞎 | 🞎 | 🞎 |
| 1. Rising from sitting | 🞎 | 🞎 | 🞎 | 🞎 | 🞎 |
| 1. Standing | 🞎 | 🞎 | 🞎 | 🞎 | 🞎 |
| 1. Bending to floor | 🞎 | 🞎 | 🞎 | 🞎 | 🞎 |
| 1. Walking on a flat surface | 🞎 | 🞎 | 🞎 | 🞎 | 🞎 |
| 1. Getting in/out of car | 🞎 | 🞎 | 🞎 | 🞎 | 🞎 |
| 1. Going shopping | 🞎 | 🞎 | 🞎 | 🞎 | 🞎 |
| 1. Putting on socks/stockings | 🞎 | 🞎 | 🞎 | 🞎 | 🞎 |
| 1. Rising from bed | 🞎 | 🞎 | 🞎 | 🞎 | 🞎 |
| 1. Taking off socks/stockings | 🞎 | 🞎 | 🞎 | 🞎 | 🞎 |
| 1. Lying in bed | 🞎 | 🞎 | 🞎 | 🞎 | 🞎 |
| 1. Getting in/out of bath | 🞎 | 🞎 | 🞎 | 🞎 | 🞎 |
| 1. Sitting | 🞎 | 🞎 | 🞎 | 🞎 | 🞎 |
| 1. Getting on/off toilet | 🞎 | 🞎 | 🞎 | 🞎 | 🞎 |
| 1. Heavy domestic duties (mowing the lawn, lifting heavy groceries) | 🞎 | 🞎 | 🞎 | 🞎 | 🞎 |
| 1. Light domestic duties (tidying a room, dusting, cooking) | 🞎 | 🞎 | 🞎 | 🞎 | 🞎 |

**SF12.v2**

The next section asks for **your views about your health**. This information will help keep track of how you feel and how well you are able to do your usual activities.

Answer every question by selecting the answer as indicated. If you are unsure about how to answer a question, please give the best answer you can.

1. In general, would you say your health is:

| Excellent | Very good | Good | Fair | Poor |
| --- | --- | --- | --- | --- |
| 🞎 | 🞎 | 🞎 | 🞎 | 🞎 |

1. The following questions are about activities you might do during a typical day. Does **your health now limit you** in these activities? If so, how much?

|  | Yes, limited a lot | Yes, limited a little | No, not limited at all |
| --- | --- | --- | --- |
| 1. Moderate activities, such as moving a table, pushing a vacuum cleaner, bowling, or playing golf | 🞎 | 🞎 | 🞎 |
| 1. Climbing several flights of stairs | 🞎 | 🞎 | 🞎 |

1. During the **past 4 weeks**, have you had any of the following problems with your work or other regular daily activities **as a result of your physical health**?

|  | All of the time | Most of the time | Some of the time | A little of the time | None of the time |
| --- | --- | --- | --- | --- | --- |
| 1. Accomplished less than you would like | 🞎 | 🞎 | 🞎 | 🞎 | 🞎 |
| 1. Were limited in the kind of work or other activities | 🞎 | 🞎 | 🞎 | 🞎 | 🞎 |

1. During the **past 4 weeks**, have you had any of the following problems with your work or other regular daily activities **as a result of any emotional problems** (such as feeling depressed or anxious)?

|  | All of the time | Most of the time | Some of the time | A little of the time | None of the time |
| --- | --- | --- | --- | --- | --- |
| 1. Accomplished less than you would like | 🞎 | 🞎 | 🞎 | 🞎 | 🞎 |
| 1. Did work or other activities less carefully than usual | 🞎 | 🞎 | 🞎 | 🞎 | 🞎 |

1. During the **past 4 weeks**, how much did **pain** interfere with your normal work (including both work outside the home and housework)?

| Not at all | A little bit | Moderately | Quite a bit | Extremely |
| --- | --- | --- | --- | --- |
| 🞎 | 🞎 | 🞎 | 🞎 | 🞎 |

1. The next questions are about how you feel and how things have been with you **during the past 4 weeks**. For each question, please give the one answer that comes closest to the way you have been feeling.

How much of the time during the **past 4 weeks**…

|  | All of the time | Most of the time | Some of the time | A little of the time | None of the time |
| --- | --- | --- | --- | --- | --- |
| 1. Have you felt calm and peaceful? | 🞎 | 🞎 | 🞎 | 🞎 | 🞎 |
| 1. Did you have a lot of energy? | 🞎 | 🞎 | 🞎 | 🞎 | 🞎 |
| 1. Have you felt downhearted and depressed? | 🞎 | 🞎 | 🞎 | 🞎 | 🞎 |

1. During the **past 4 weeks**, how much of the time has your **physical health or emotional problems** interfered with your social activities (like visiting friends, relatives, etc.)?

| All of the  time | Most of  the time | Some of  the time | A little of  the time | None of  the time |
| --- | --- | --- | --- | --- |
| 🞎 | 🞎 | 🞎 | 🞎 | 🞎 |

**Beliefs Questionnaire**

| Here are some of the things which other patients have told us about their pain. For each statement please circle any number from 0 to 6 to say how much physical activities such as bending, lifting, walking or driving affect or would affect **your knee pain** | | | | | | | | | |
| --- | --- | --- | --- | --- | --- | --- | --- | --- | --- |
|  | Completely disagree | | | Unsure | | | Completely agree | | |
| 1. My pain was caused by physical activity | 0 | 1 | 2 | | 3 | 4 | | 5 | 6 |
| 1. Physical activity makes my pain worse | 0 | 1 | 2 | | 3 | 4 | | 5 | 6 |
| 1. Physical activity might harm my knee | 0 | 1 | 2 | | 3 | 4 | | 5 | 6 |
| 1. I should not do physical activities which (might) make my pain worse | 0 | 1 | 2 | | 3 | 4 | | 5 | 6 |
| 1. I cannot do physical activities which (might) make my pain worse | 0 | 1 | 2 | | 3 | 4 | | 5 | 6 |

**Beliefs Questionnaire**

|  |
| --- |

The next question is a series of statements aimed to help us understand your beliefs about physical pain.

Please indicate how much you agree with each of the following statements about **your** **pain** problem by using the following scale.

*The rating scale is as follows:*

0 Very untrue for me

1 Somewhat untrue for me

2 Neither true nor untrue for me (or does not apply)

3 Somewhat true for me

4 Very true for me

**Please circle your response**

| 1. There is little I can do to ease my pain | 0 | 1 | 2 | 3 | 4 |
| --- | --- | --- | --- | --- | --- |
| 1. My pain does not stop me from leading a physically active life | 0 | 1 | 2 | 3 | 4 |
| 1. The pain I feel is a sign that damage is being done | 0 | 1 | 2 | 3 | 4 |
| 1. There is a connection between my emotions and my pain level | 0 | 1 | 2 | 3 | 4 |
| 1. I will probably always have to take pain medications | 0 | 1 | 2 | 3 | 4 |
| 1. When I am hurting, I deserve to be treated with care and concern | 0 | 1 | 2 | 3 | 4 |
| 1. I trust that doctors can cure my pain | 0 | 1 | 2 | 3 | 4 |

**DASS Scale**

Please read each statement and circle a number 0, 1, 2 or 3 which indicates how much the statement applied to you **over the past week**. There are no right or wrong answers. Do not spend too much time on any statement.

*The rating scale is as follows:*

0 Did not apply to me at all

1 Applied to me to some degree, or some of the time

2 Applied to me to a considerable degree, or a good part of time

3 Applied to me very much, or most of the time

**Please circle your response**

| 1. I found it hard to wind down | 0 | 1 | 2 | 3 |
| --- | --- | --- | --- | --- |
| 1. I was aware of dryness of my mouth | 0 | 1 | 2 | 3 |
| 1. I couldn't seem to experience any positive feeling at all | 0 | 1 | 2 | 3 |
| 1. I experienced breathing difficulty (eg, excessively rapid breathing, breathlessness in the absence of physical exertion) | 0 | 1 | 2 | 3 |
| 1. I found it difficult to work up the initiative to do things | 0 | 1 | 2 | 3 |
| 1. I tended to over-react to situations | 0 | 1 | 2 | 3 |
| 1. I experienced trembling (eg, in the hands) | 0 | 1 | 2 | 3 |
| 1. I felt that I was using a lot of nervous energy | 0 | 1 | 2 | 3 |
| 1. I was worried about situations in which I might panic and make a fool of myself | 0 | 1 | 2 | 3 |
| 1. I felt that I had nothing to look forward to | 0 | 1 | 2 | 3 |
| 1. I found myself getting agitated | 0 | 1 | 2 | 3 |
| 1. I found it difficult to relax | 0 | 1 | 2 | 3 |
| 1. I felt down-hearted and blue | 0 | 1 | 2 | 3 |

*The rating scale is as follows:*

0 Did not apply to me at all

1 Applied to me to some degree, or some of the time

2 Applied to me to a considerable degree, or a good part of time

3 Applied to me very much, or most of the time

**Please circle your response**

| 1. I was intolerant of anything that kept me from getting on with what I was doing | 0 | 1 | 2 | 3 |
| --- | --- | --- | --- | --- |
| 1. I felt I was close to panic | 0 | 1 | 2 | 3 |
| 1. I was unable to become enthusiastic about anything | 0 | 1 | 2 | 3 |
| 1. I felt I wasn't worth much as a person | 0 | 1 | 2 | 3 |
| 1. I felt that I was rather touchy | 0 | 1 | 2 | 3 |
| 1. I was aware of the action of my heart in the absence of physical exertion (e.g., sense of heart rate increase, heart missing a beat) | 0 | 1 | 2 | 3 |
| 1. I felt scared without any good reason | 0 | 1 | 2 | 3 |
| 1. I felt that life was meaningless | 0 | 1 | 2 | 3 |

**Smoking Status**

The next questions will ask you about your tobacco use.

1. **Have you EVER been a smoker?**

🞎 Smoker (please go to question 3)

🞎 Ex-Smoker (continue)

🞎 Never smoked (please go to next section ‘’The Active Australia Survey’)

1. **IF EX-SMOKER, approximately when did you cease smoking?**

🞎 Within the last 3 months

🞎 3-12 months ago

🞎 1-2 years ago

🞎 3-5 years ago

🞎 More than 5 years ago

🞎 Don’t know

1. **Have you smoked any tobacco in the LAST 4 WEEKS? (this can include cigarettes, roll your own, pipes, cigars or any other tobacco products)**

🞎 Yes (go to question 4)

🞎 No (please go to page 12 ‘The Active Australia Survey’)

1. **Do you CURRENTLY SMOKE 10 or more cigarettes (10 roll your own, 2 cigars, or 1 pipe) a day?**

🞎 Yes

🞎 No

🞎 Don’t know

1. **Do you CURRENTLY SMOKE within half an hour of waking**

🞎 Yes

🞎 No

🞎 Don’t know

**The Active Australia Survey**

The next questions are about any physical activity that you may have done in the last week. If you **DID NOT** do an activity, please write ‘0’ in the box

1. In the **last week**, how many times have you **walked continuously**, for at **least 10 minutes,** for recreation, exercise or to get to or from places? (This must be continuous walking, i.e. for at least 10 minutes without stopping)


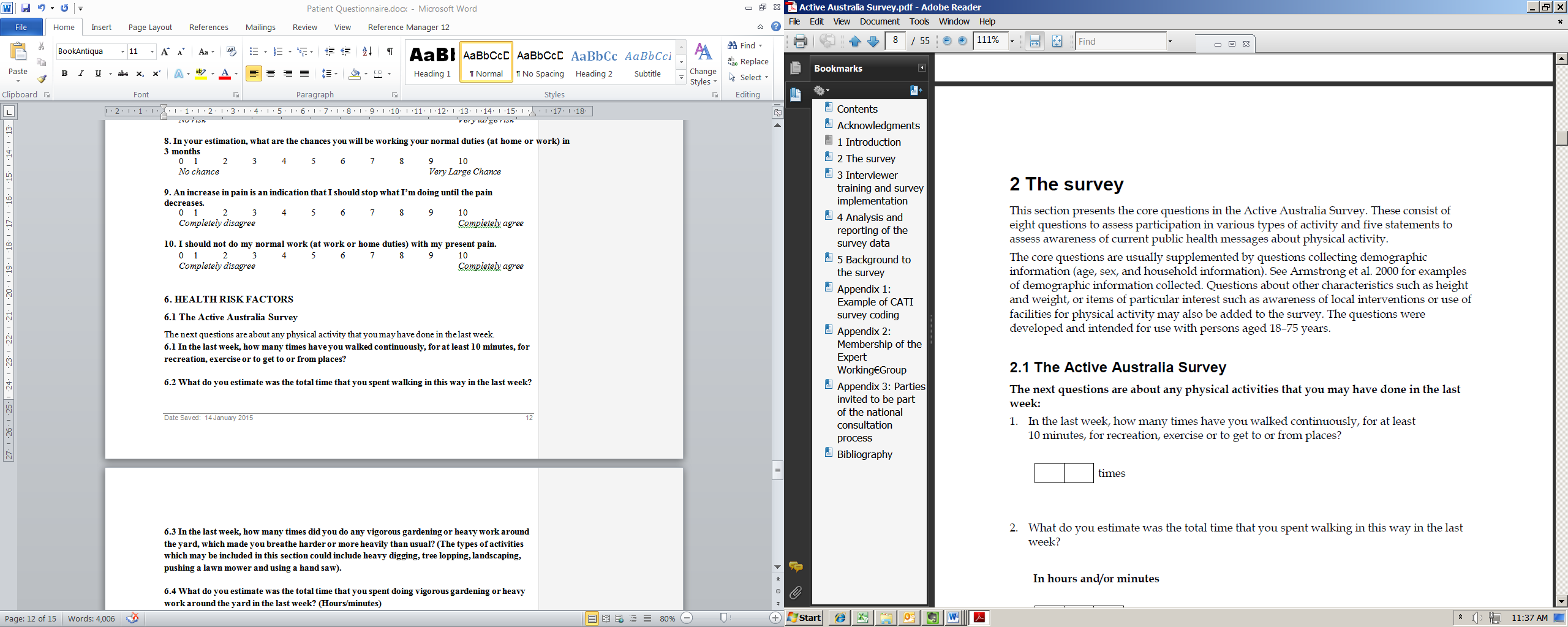


1. What do you estimate was the **total time** that you **spent walking** in this way in the last week?

In hours and/or minutes


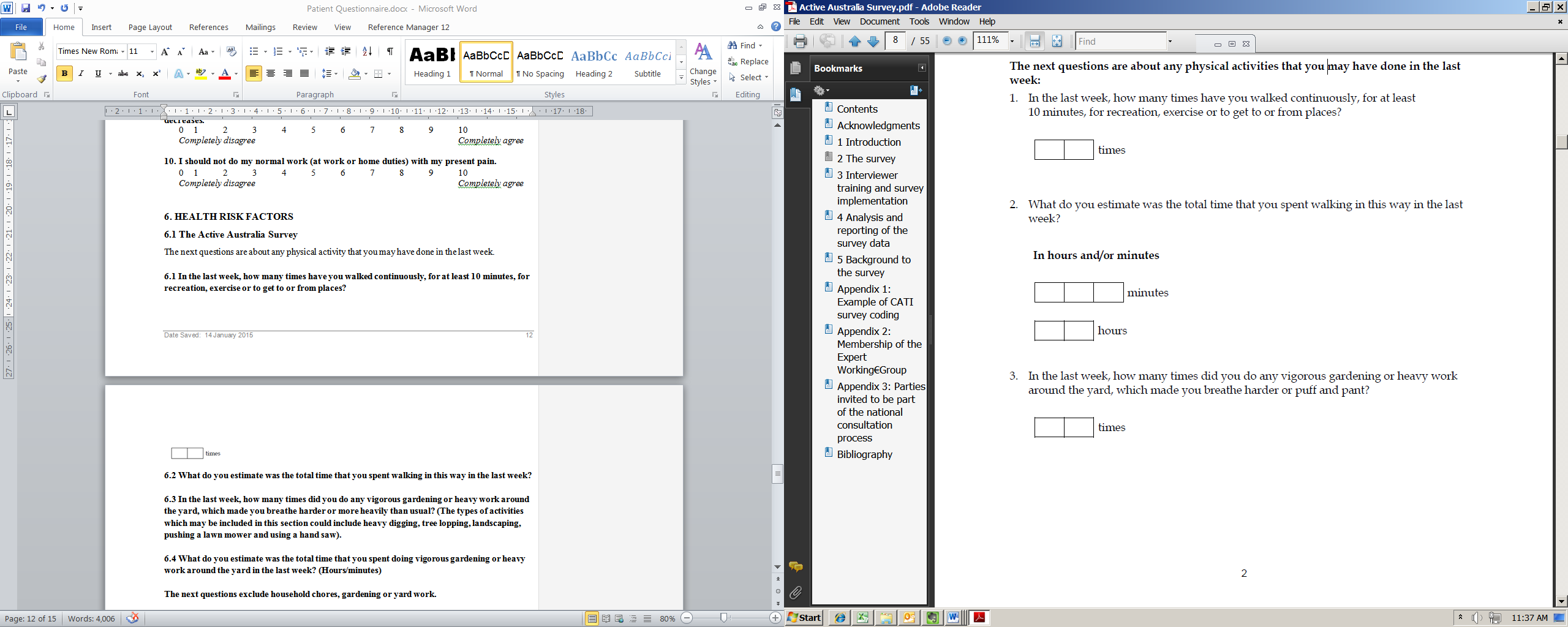


1. In the **last week**, how many times did you do any **vigorous gardening or heavy work around the yard**, which made you breathe harder or more heavily than usual? (The types of activities which may be included in this section could include heavy digging, tree lopping, landscaping, pushing a lawn mower and using a hand saw).


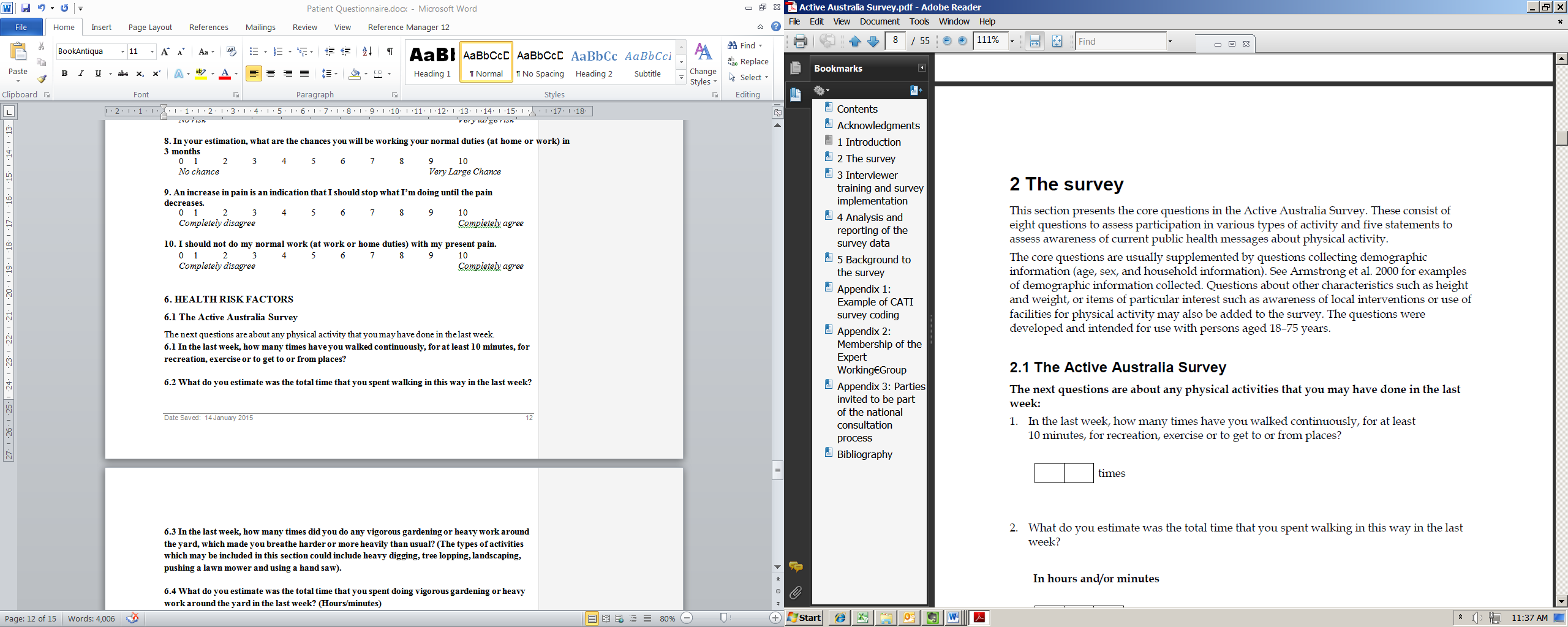


1. What do you estimate was the **total time** that you **spent doing vigorous gardening or heavy work around the yard** in the last week? (Hours/minutes)

In hours and/or minutes


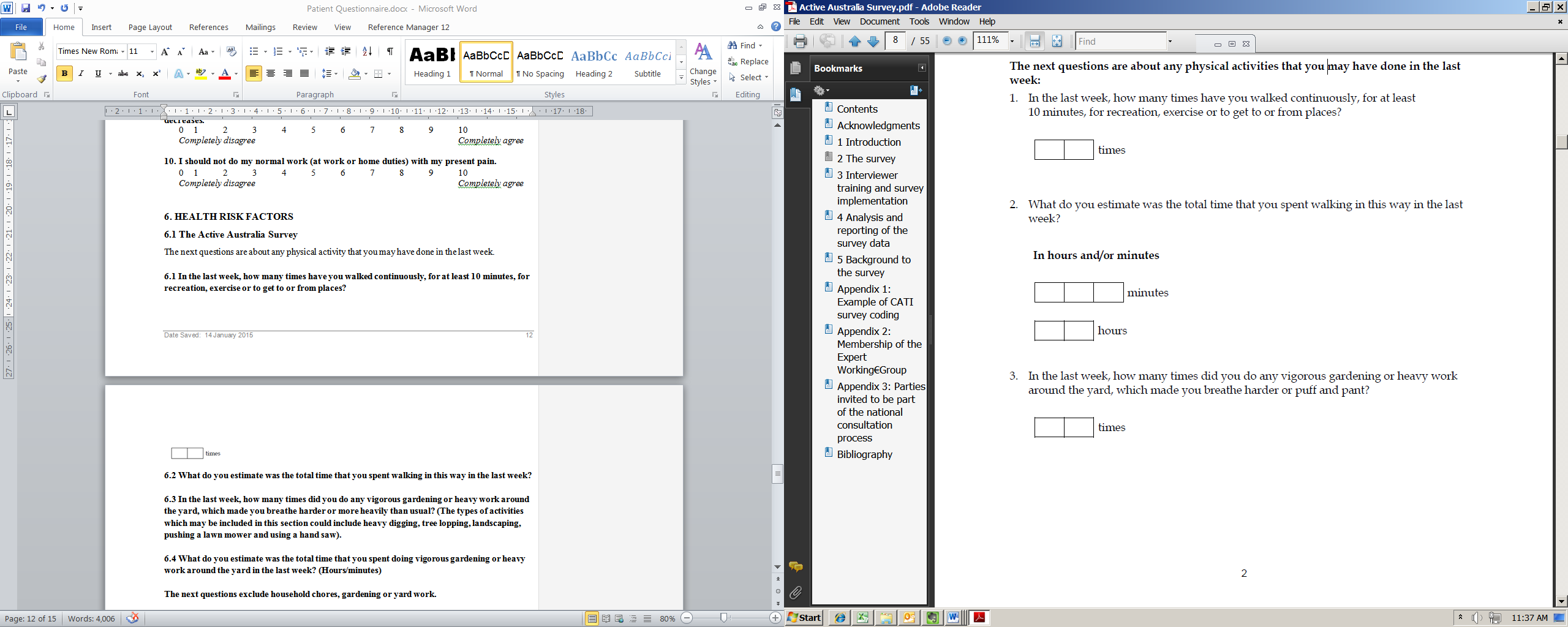


The next questions exclude household chores, gardening or yard work.

1. In the **last week**, how many times did you do any **vigorous physical activity** which made you breathe harder or more heavily than usual? (E.g. jogging, cycling, aerobics, competitive tennis, football, hockey, squash, hiking, weight lifting, boxing, basketball, netball, step aerobics)


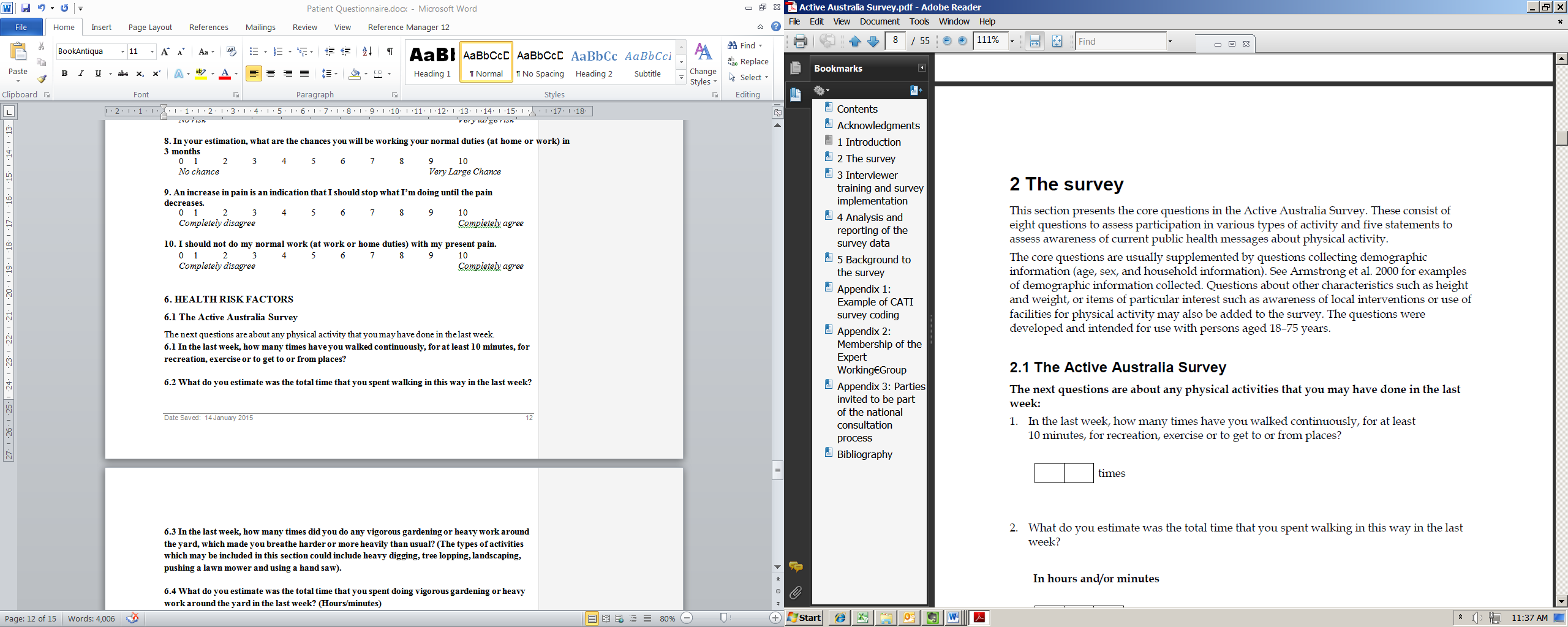


1. What do you estimate was the **total time** that you **spent doing this vigorous physical activity** in the last week?

In hours and/or minutes


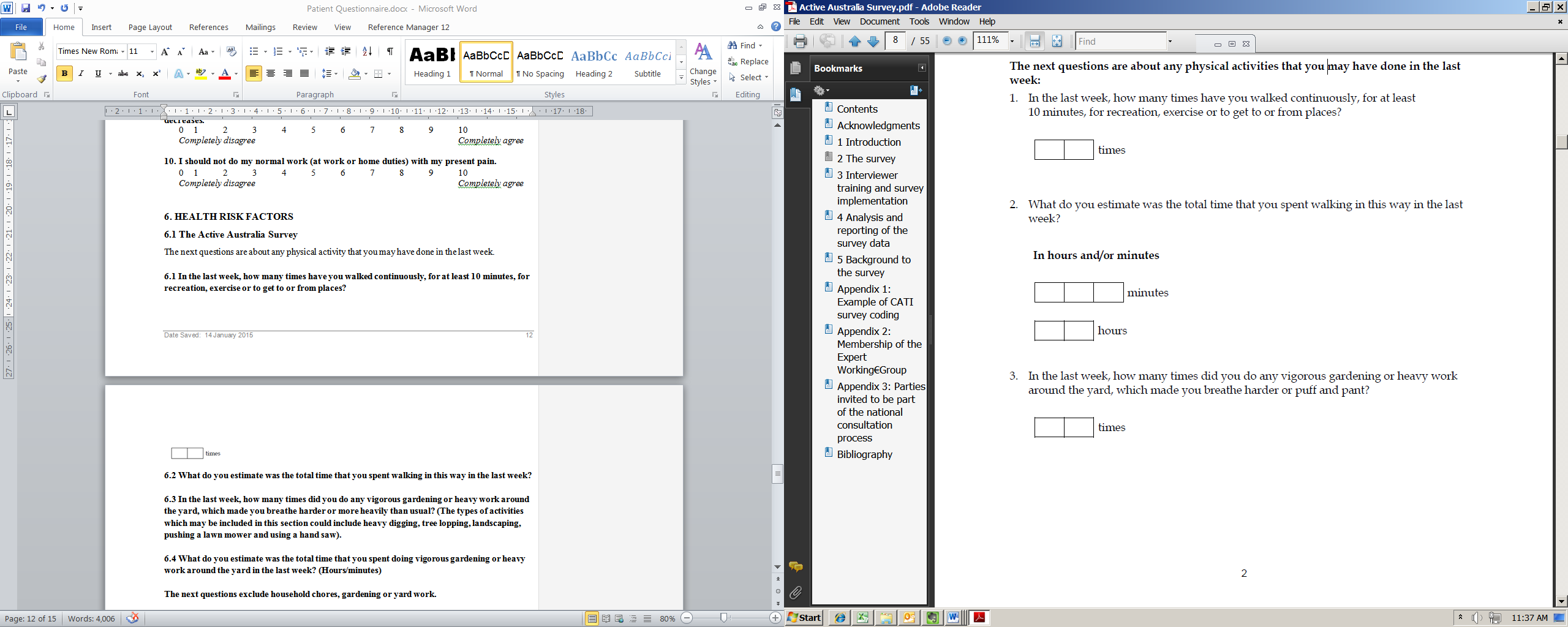


1. In the **last week**, how many times did you do any other more **moderate physical activities** that you have not already mentioned? (E.g. gentle swimming, social tennis, golf, dancing, table tennis, canoeing, cricket)


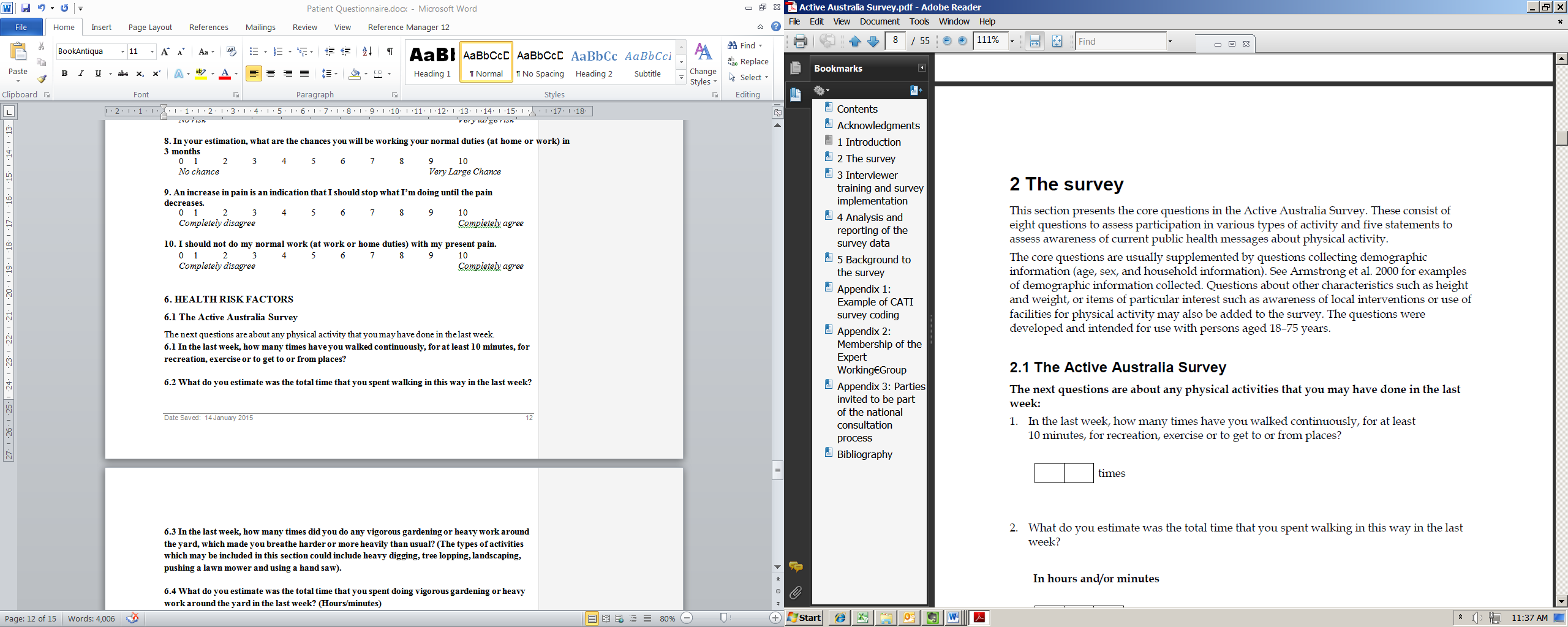


1. What do you estimate was the **total time** that you **spent doing these activities** in the last week?

In hours and/or minutes


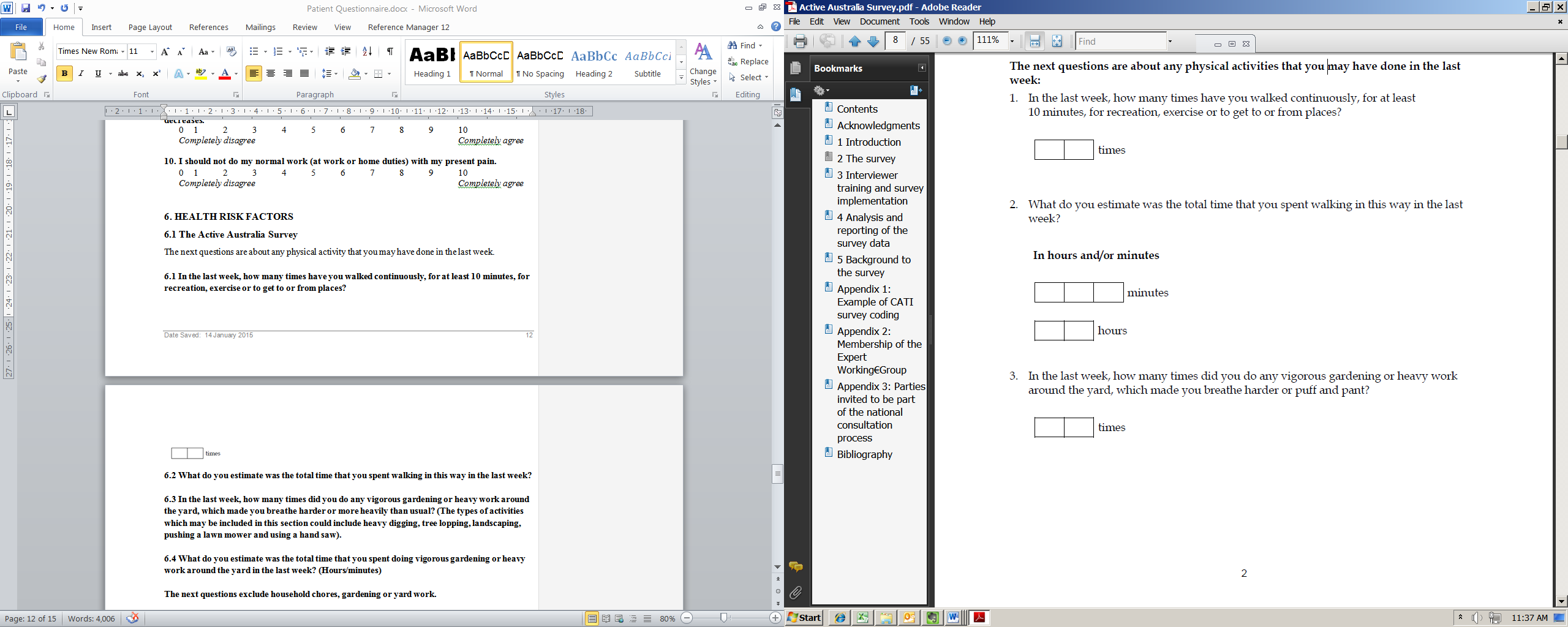


**Food Frequency Questionnaire**

The next questions ask you about your eating habits.

**Vegetables**

1. **How many serves of vegetables do you eat in a usual day?** *(*1 serve of vegetables is 75g or 1/2 cup cooked vegetables OR 75g or 1/2 cup cooked dried beans, peas or lentils OR 1 cup salad vegetables OR 1 potato*).*

🞎 0

🞎 1

🞎 2

🞎 3

🞎 4

🞎 5 or more

**Fruit**

1. **How many serves of fruit do you eat in a usual day?** *(*1 serve of fruit is: 1 medium apple, banana, orange, pear, OR 2 small pieces apricots, kiwi fruit, plums OR 1 cup diced pieces OR canned fruit OR 1/2 cup juice*).*

🞎 0

🞎 1

🞎 2 or more

**Meat**

1. **How often do you eat red meat, such as beef or lamb?** (Include all steaks, chops, roasts, mince, stir fries and casseroles. Do not include pork or chicken*).*

🞎 More than 1 time per day

🞎 Once per day

🞎 More than once a week

🞎 Once per week

🞎 More than once per month

🞎 Rarely or never

**Processed Foods**

1. **How often do you eat processed meat products such as sausages, frankfurts, devon, salami, meat pies, bacon or ham?**

🞎 More than 1 time per day

🞎 Once per day

🞎 More than once a week

🞎 Once per week

🞎 More than once per month

🞎 Rarely or never

1. **How often do you eat potato crisps or other salty snacks?** *(*such as Twisties or corn chips*)*

🞎 More than 1 time per day

🞎 Once per day

🞎 More than once per week

🞎 Once per week

🞎 More than once per month

🞎 Rarely or never

1. **How often do you have meals or snacks such as burgers, pizza, chicken or chips from places like McDonalds, Hungry Jacks, KFC, Red Rooster?**

🞎 More than 1 time per day

🞎 Once per day

🞎 More than once a week

🞎 Once per week

🞎 More than once per month

🞎 Rarely or never

1. **How often do you have snack foods such as sweet or savoury biscuits, cakes, doughnuts or muesli bars?**

🞎 More than 1 time per day

🞎 Once per day

🞎 More than once a week

🞎 Once per week

🞎 More than once per month

🞎 Rarely or never

1. **How often do you eat confectionary, such as lollies and chocolate?**

🞎 More than 1 time per day

🞎 Once per day

🞎 More than once a week

🞎 Once per week

🞎 More than once per month

🞎 Rarely or never

**Breads and cereals**

1. **How often do you usually eat bread?** *(*Include bread rolls, flat breads, crumpets, bagels, English or bread type muffins*)*

🞎 More than 1 time per day

🞎 Once per day

🞎 More than once a week

🞎 Once per week

🞎 More than once per month

🞎 Rarely or never

1. **How often do you eat pasta, rice, or noodles?**

🞎 More than 1 time per day

🞎 Once per day

🞎 More than once a week

🞎 Once per week

🞎 More than once per month

🞎 Rarely or never

**Drinks**

1. **How many cups of milk do you usually drink a day?** *(*1 cup = 250mL*)*

🞎 More than one cup per day

🞎 Around one cup per day

🞎 One cup per week

🞎 One cup more than once per week

🞎 One cup more than once per month

🞎 Rarely or never

1. **How many cups of soft drink, cordials or sports drink such as lemonade or Gatorade do you usually drink in a day?** *(*1 cup = 250mL, 1 can of soft drink = 1.5 cups, 1 x 500mL bottle of Gatorade = 2 cups*)*

🞎 More than one cup per day

🞎 Around one cup per day

🞎 One cup per week

🞎 One cup more than once per week

🞎 One cup more than once per month

🞎 Rarely or never

1. **How many cups of water do you usually drink a day?** *(*1 cup = 250mL*)*

🞎 More than one cup per day

🞎 Around one cup per day

🞎 One cup per week

🞎 One cup more than once per week

🞎 One cup more than once per month

🞎 Rarely or never

**Food Security**

1. **In the last 12 months were there any times that you ran out of food and couldn’t afford to buy more?**

🞎 Yes

🞎 No

🞎 Don’t know

**Alcohol Consumption**

|  |  |
| --- | --- |

For this section, alcoholic drinks are measured in terms of a standard drink. A standard drink is equal to one middy of beer, one schooner of light beer, one small glass of wine or one pub-sized nip of spirits.

1. **How often do you have a drink containing alcohol?**

🞎 Never (finished questionnaire)

🞎 Monthly or less

🞎 2 to 4 times a month

🞎 2 to 3 times a week

🞎 4 or more times a week

1. **How many STANDARD DRINKS would you have on a typical drinking day?** (A standard drink is equal to one middy of beer, one schooner of light beer, one small glass of wine or one pub-sized nip of spirits)

🞎 1-2

🞎 3-4

🞎 5-6

🞎 7-9

🞎 10 or more

1. **How often would you have 4 OR MORE DRINKS on any occasion?**

🞎 Never

🞎 Less than monthly

🞎 Monthly

🞎 Weekly

🞎 Daily or almost daily

**Thank you! That is the end of the questionnaire**

# Appendix 2 – Patient Questionnaire (Week 6 and 26)

**Knee Pain Questionnaire**

**Name:** ____________________________

**Date:** ____/_____/_____

**Instructions**

Please complete all questions. The need to complete some smoking status and alcohol consumption questions will depend on your previous answers. Please read instructions carefully.

**Pain medication**

**Are you currently taking any medications for your knee pain?**

🞎 Yes – please specify below 🞎 No – continue to the next question

| **Knee pain medication name and strength (mg)** | **Dose (number of tablets per day)** | **Length of time you have been taking this medication** (in weeks, months and/or years e.g. 2 years 6 months) |
| --- | --- | --- |
|  |  |  |
|  |  |  |
|  |  |  |
|  |  |  |
|  |  |  |

**Health service utilisation for your knee pain**

Did you use any health services in the **last 6 weeks** for your knee pain? This includes seeing a general practitioner (GP), a physiotherapist or dietitian, any hospital admissions or use of any community health or other services?

🞎 Yes – please specify below 🞎 No – continue to the next question

| **Type of service** | **Number of sessions** | **Estimated out of pocket cost** |
| --- | --- | --- |
|  |  |  |
|  |  |  |
|  |  |  |
|  |  |  |
|  |  |  |

**Since we last spoke to you (approximately 6 weeks ago) have you had any new medical conditions or an exacerbation of another existing condition?**

If yes, what was it: _______________________________________________________

When did it start (date): ______________ How long did it last (days):_______________

**Do you know how much you currently weigh?** If you do not know please provide an estimate.

________ Kilograms / stones / pounds (please circle unit of measurement)

**Pain Characteristics**

1. **How would you your average knee pain over the LAST WEEK on a scale from 0 to 10, where 0 is no pain and 10 is the worst possible pain. Please circle a number below.**

| **0** | **1** | **2** | **3** | **4** | **5** | **6** | **7** | **8** | **9** | **10** |
| --- | --- | --- | --- | --- | --- | --- | --- | --- | --- | --- |

No pain Worst possible pain

1. **Compared to when this program first started (approximately 6 weeks ago), how would you describe your knee these days?**

| **- 5** | **- 4** | **- 3** | **- 2** | **- 1** | **0** | **1** | **2** | **3** | **4** | **5** |
| --- | --- | --- | --- | --- | --- | --- | --- | --- | --- | --- |

Vastly worse Unchanged Completely

Recovered

1. **How many days, since starting the program (approximately 6 weeks ago), did your knee pain force you to cut down on the things you usually do, for more than half a day?** If your knee pain didn’t affect your usual activities please write “0”

______________________Days

1. **In the last 6 weeks how many days off your normal paid work did you have due to your knee pain?** If you are not employed or did not have any days off please write 0

______________________Days

1. **During the PAST WEEK, how would you rate your sleep quality overall?**

| 🞎 Very good | 🞎 Fairly good | 🞎 Fairly bad | 🞎 Very bad |
| --- | --- | --- | --- |

**WOMAC**

1. The following questions concern the amount of pain you are currently experiencing in your knees. For each situation, please enter the amount of pain you have experienced in the **past 48 hours**

|  | None | Mild | Moderate | Severe | Extreme |
| --- | --- | --- | --- | --- | --- |
| 1. Walking on a flat surface | 🞎 | 🞎 | 🞎 | 🞎 | 🞎 |
| 1. Going up or down stairs | 🞎 | 🞎 | 🞎 | 🞎 | 🞎 |
| 1. At night while in bed | 🞎 | 🞎 | 🞎 | 🞎 | 🞎 |
| 1. Sitting or lying | 🞎 | 🞎 | 🞎 | 🞎 | 🞎 |
| 1. Standing upright | 🞎 | 🞎 | 🞎 | 🞎 | 🞎 |

1. Please describe the level of pain you have experienced in the **past 48 hours** for each one of your knees

|  | None | Mild | Moderate | Severe | Extreme |
| --- | --- | --- | --- | --- | --- |
| Right knee | 🞎 | 🞎 | 🞎 | 🞎 | 🞎 |
| Left knee | 🞎 | 🞎 | 🞎 | 🞎 | 🞎 |

1. How severe is your stiffness after first awakening in the morning?

| None | Mild | Moderate | Severe | Extreme |
| --- | --- | --- | --- | --- |
| 🞎 | 🞎 | 🞎 | 🞎 | 🞎 |

1. How severe is your stiffness after sitting, lying, or resting later in the day?

| None | Mild | Moderate | Severe | Extreme |
| --- | --- | --- | --- | --- |
| 🞎 | 🞎 | 🞎 | 🞎 | 🞎 |

1. The following questions concern **your physical function**. By this we mean your ability to move around and to look after yourself. For each of the following activities, please indicate the **degree of difficulty** you have experienced in the **last 48 hours**, in your knees.

What degree of difficulty do you have with:

|  | None | Mild | Moderate | Severe | Extreme |
| --- | --- | --- | --- | --- | --- |
| 1. Going down stairs | 🞎 | 🞎 | 🞎 | 🞎 | 🞎 |
| 1. Going up stairs | 🞎 | 🞎 | 🞎 | 🞎 | 🞎 |
| 1. Rising from sitting | 🞎 | 🞎 | 🞎 | 🞎 | 🞎 |
| 1. Standing | 🞎 | 🞎 | 🞎 | 🞎 | 🞎 |
| 1. Bending to floor | 🞎 | 🞎 | 🞎 | 🞎 | 🞎 |
| 1. Walking on a flat surface | 🞎 | 🞎 | 🞎 | 🞎 | 🞎 |
| 1. Getting in/out of car | 🞎 | 🞎 | 🞎 | 🞎 | 🞎 |
| 1. Going shopping | 🞎 | 🞎 | 🞎 | 🞎 | 🞎 |
| 1. Putting on socks/stockings | 🞎 | 🞎 | 🞎 | 🞎 | 🞎 |
| 1. Rising from bed | 🞎 | 🞎 | 🞎 | 🞎 | 🞎 |
| 1. Taking off socks/stockings | 🞎 | 🞎 | 🞎 | 🞎 | 🞎 |
| 1. Lying in bed | 🞎 | 🞎 | 🞎 | 🞎 | 🞎 |
| 1. Getting in/out of bath | 🞎 | 🞎 | 🞎 | 🞎 | 🞎 |
| 1. Sitting | 🞎 | 🞎 | 🞎 | 🞎 | 🞎 |
| 1. Getting on/off toilet | 🞎 | 🞎 | 🞎 | 🞎 | 🞎 |
| 1. Heavy domestic duties (mowing the lawn, lifting heavy groceries) | 🞎 | 🞎 | 🞎 | 🞎 | 🞎 |
| 1. Light domestic duties (tidying a room, dusting, cooking) | 🞎 | 🞎 | 🞎 | 🞎 | 🞎 |

**SF12.v2**

The next section asks for **your views about your health**. This information will help keep track of how you feel and how well you are able to do your usual activities.

Answer every question by selecting the answer as indicated. If you are unsure about how to answer a question, please give the best answer you can.

1. In general, would you say your health is:

| Excellent | Very good | Good | Fair | Poor |
| --- | --- | --- | --- | --- |
| 🞎 | 🞎 | 🞎 | 🞎 | 🞎 |

1. The following questions are about activities you might do during a typical day. Does **your health now limit you** in these activities? If so, how much?

|  | Yes, limited a lot | Yes, limited a little | No, not limited at all |
| --- | --- | --- | --- |
| 1. Moderate activities, such as moving a table, pushing a vacuum cleaner, bowling, or playing golf | 🞎 | 🞎 | 🞎 |
| 1. Climbing several flights of stairs | 🞎 | 🞎 | 🞎 |

1. During the **past 4 weeks**, have you had any of the following problems with your work or other regular daily activities **as a result of your physical health**?

|  | All of the time | Most of the time | Some of the time | A little of the time | None of the time |
| --- | --- | --- | --- | --- | --- |
| 1. Accomplished less than you would like | 🞎 | 🞎 | 🞎 | 🞎 | 🞎 |
| 1. Were limited in the kind of work or other activities | 🞎 | 🞎 | 🞎 | 🞎 | 🞎 |

1. During the **past 4 weeks**, have you had any of the following problems with your work or other regular daily activities **as a result of any emotional problems** (such as feeling depressed or anxious)?

|  | All of the time | Most of the time | Some of the time | A little of the time | None of the time |
| --- | --- | --- | --- | --- | --- |
| 1. Accomplished less than you would like | 🞎 | 🞎 | 🞎 | 🞎 | 🞎 |
| 1. Did work or other activities less carefully than usual | 🞎 | 🞎 | 🞎 | 🞎 | 🞎 |

1. During the **past 4 weeks**, how much did **pain** interfere with your normal work (including both work outside the home and housework)?

| Not at all | A little bit | Moderately | Quite a bit | Extremely |
| --- | --- | --- | --- | --- |
| 🞎 | 🞎 | 🞎 | 🞎 | 🞎 |

1. The next questions are about how you feel and how things have been with you **during the past 4 weeks**. For each question, please give the one answer that comes closest to the way you have been feeling.

How much of the time during the **past 4 weeks**…

|  | All of the time | Most of the time | Some of the time | A little of the time | None of the time |
| --- | --- | --- | --- | --- | --- |
| 1. Have you felt calm and peaceful? | 🞎 | 🞎 | 🞎 | 🞎 | 🞎 |
| 1. Did you have a lot of energy? | 🞎 | 🞎 | 🞎 | 🞎 | 🞎 |
| 1. Have you felt downhearted and depressed? | 🞎 | 🞎 | 🞎 | 🞎 | 🞎 |

1. During the **past 4 weeks**, how much of the time has your **physical health or emotional problems** interfered with your social activities (like visiting friends, relatives, etc.)?

| All of the  time | Most of  the time | Some of  the time | A little of  the time | None of  the time |
| --- | --- | --- | --- | --- |
| 🞎 | 🞎 | 🞎 | 🞎 | 🞎 |

**Beliefs Questionnaire**

|  |
| --- |

The next question is a series of statements aimed to help us understand your beliefs about physical pain.

Please indicate how much you agree with each of the following statements about **your** **pain** problem by using the following scale.

*The rating scale is as follows:*

0 Very untrue for me

1 Somewhat untrue for me

2 Neither true nor untrue for me (or does not apply)

3 Somewhat true for me

4 Very true for me

**Please circle your response**

| 1. There is little I can do to ease my pain | 0 | 1 | 2 | 3 | 4 |
| --- | --- | --- | --- | --- | --- |
| 1. My pain does not stop me from leading a physically active life | 0 | 1 | 2 | 3 | 4 |
| 1. The pain I feel is a sign that damage is being done | 0 | 1 | 2 | 3 | 4 |
| 1. There is a connection between my emotions and my pain level | 0 | 1 | 2 | 3 | 4 |
| 1. I will probably always have to take pain medications | 0 | 1 | 2 | 3 | 4 |
| 1. When I am hurting, I deserve to be treated with care and concern | 0 | 1 | 2 | 3 | 4 |
| 1. I trust that doctors can cure my pain | 0 | 1 | 2 | 3 | 4 |

**Smoking Status**

The next questions will ask you about your tobacco use.

1. **Have you EVER been a smoker?**

🞎 Smoker (please go to question 3)

🞎 Ex-Smoker (continue)

🞎 Never smoked (please go to page 12 ‘The Active Australia Survey’)

1. **IF EX-SMOKER, approximately when did you cease smoking?**

🞎 Within the last 3 months

🞎 3-12 months ago

🞎 1-2 years ago

🞎 3-5 years ago

🞎 More than 5 years ago

🞎 Don’t know

1. **Have you smoked any tobacco in the LAST 4 WEEKS? (this can include cigarettes, roll your own, pipes, cigars or any other tobacco products)**

🞎 Yes (go to question 4)

🞎 No (please go to page 12 ‘The Active Australia Survey’)

1. **Do you CURRENTLY SMOKE 10 or more cigarettes (10 roll your own, 2 cigars, or 1 pipe) a day?**

🞎 Yes

🞎 No

🞎 Don’t know

1. **Do you CURRENTLY SMOKE within half an hour of waking**

🞎 Yes

🞎 No

🞎 Don’t know

**The Active Australia Survey**

The next questions are about any physical activity that you may have done in the last week. If you **DID NOT** do an activity, please write ‘0’ in the box

1. In the **last week**, how many times have you **walked continuously**, for at **least 10 minutes,** for recreation, exercise or to get to or from places? (This must be continuous walking, i.e. for at least 10 minutes without stopping)


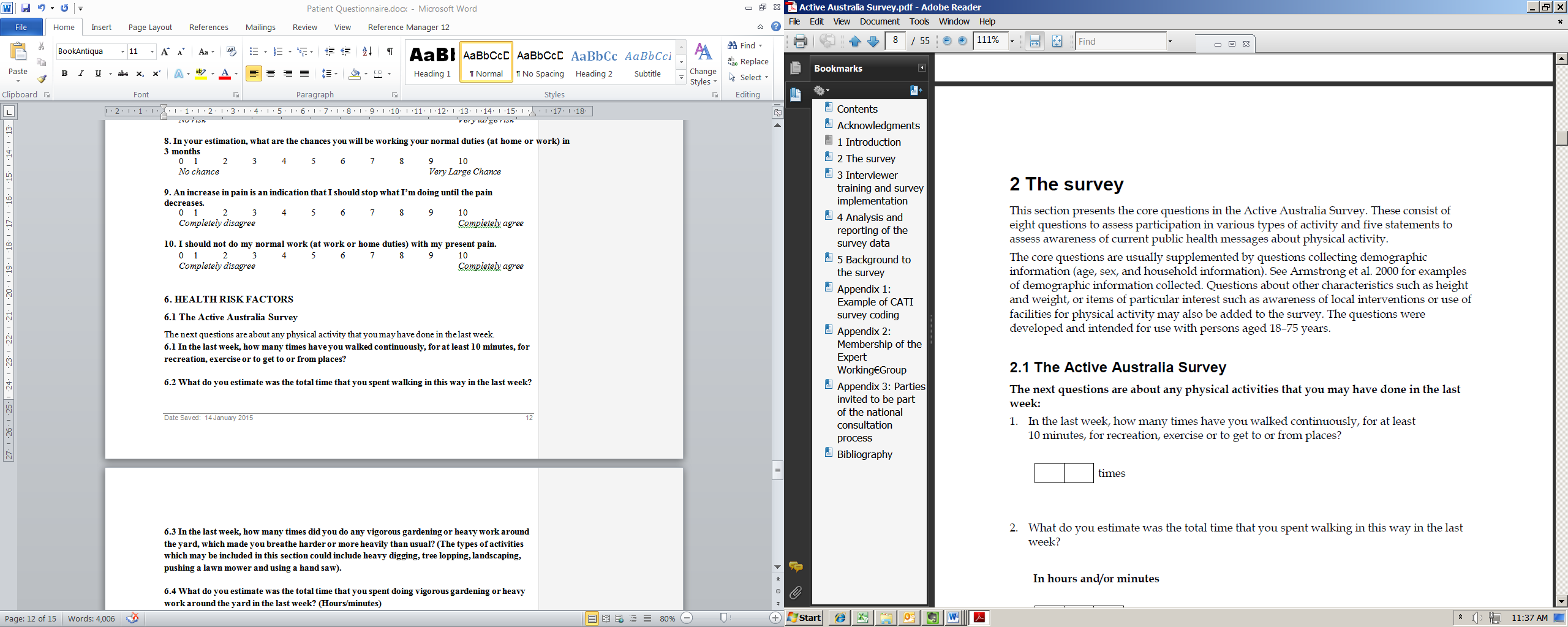


1. What do you estimate was the **total time** that you **spent walking** in this way in the last week?

In hours and/or minutes


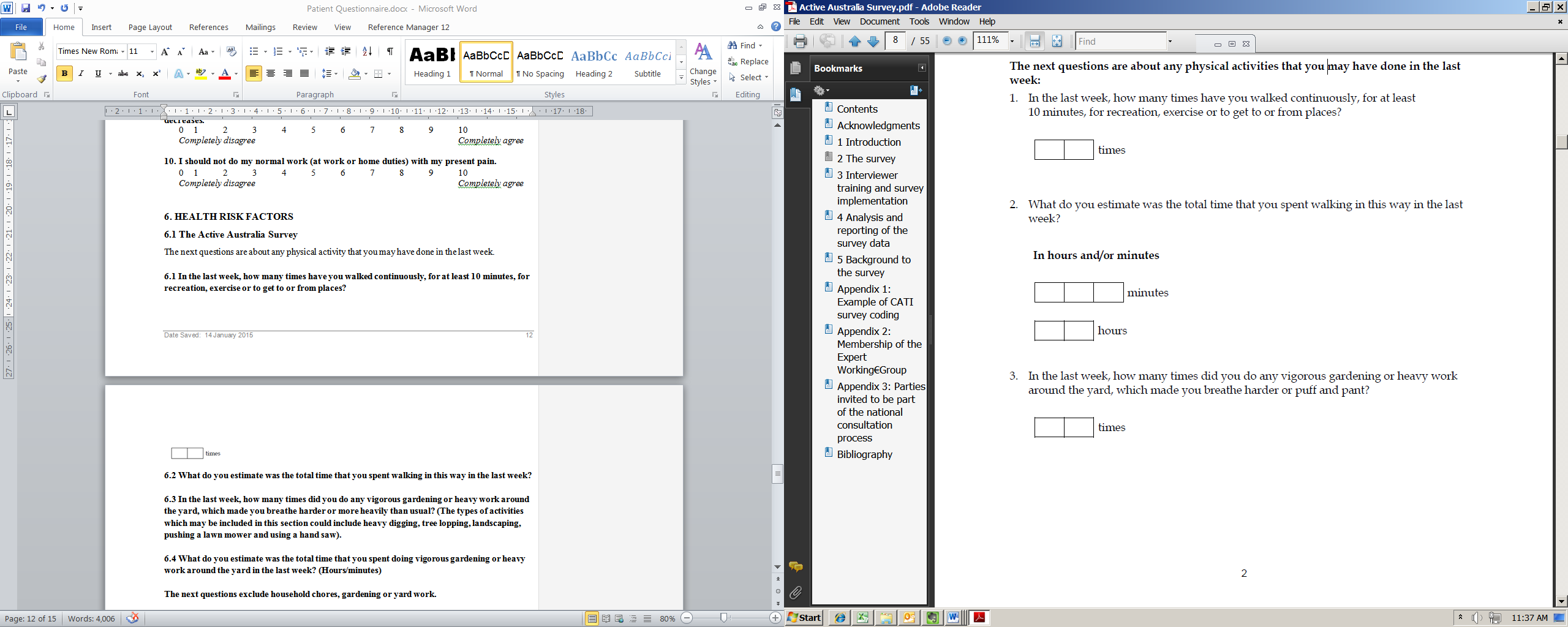


1. In the **last week**, how many times did you do any **vigorous gardening or heavy work around the yard**, which made you breathe harder or more heavily than usual? (The types of activities which may be included in this section could include heavy digging, tree lopping, landscaping, pushing a lawn mower and using a hand saw).


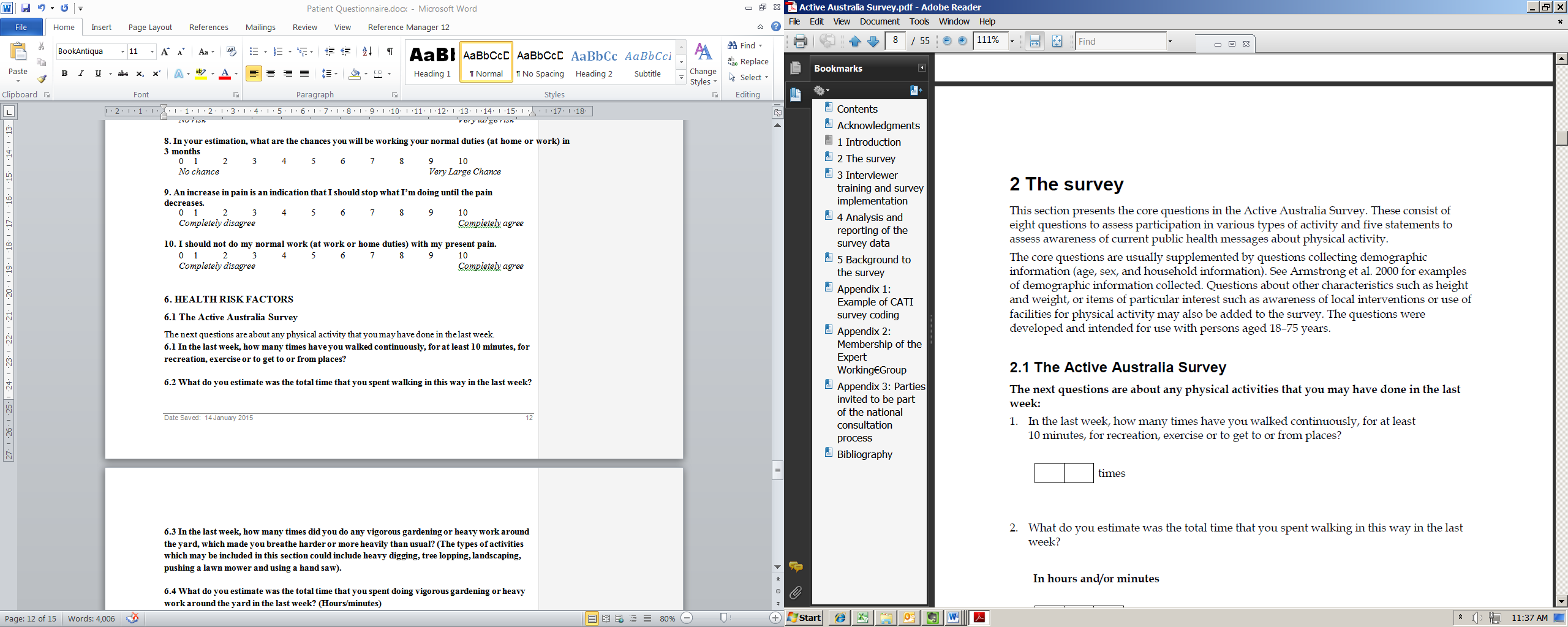


1. What do you estimate was the **total time** that you **spent doing vigorous gardening or heavy work around the yard** in the last week? (Hours/minutes)

In hours and/or minutes


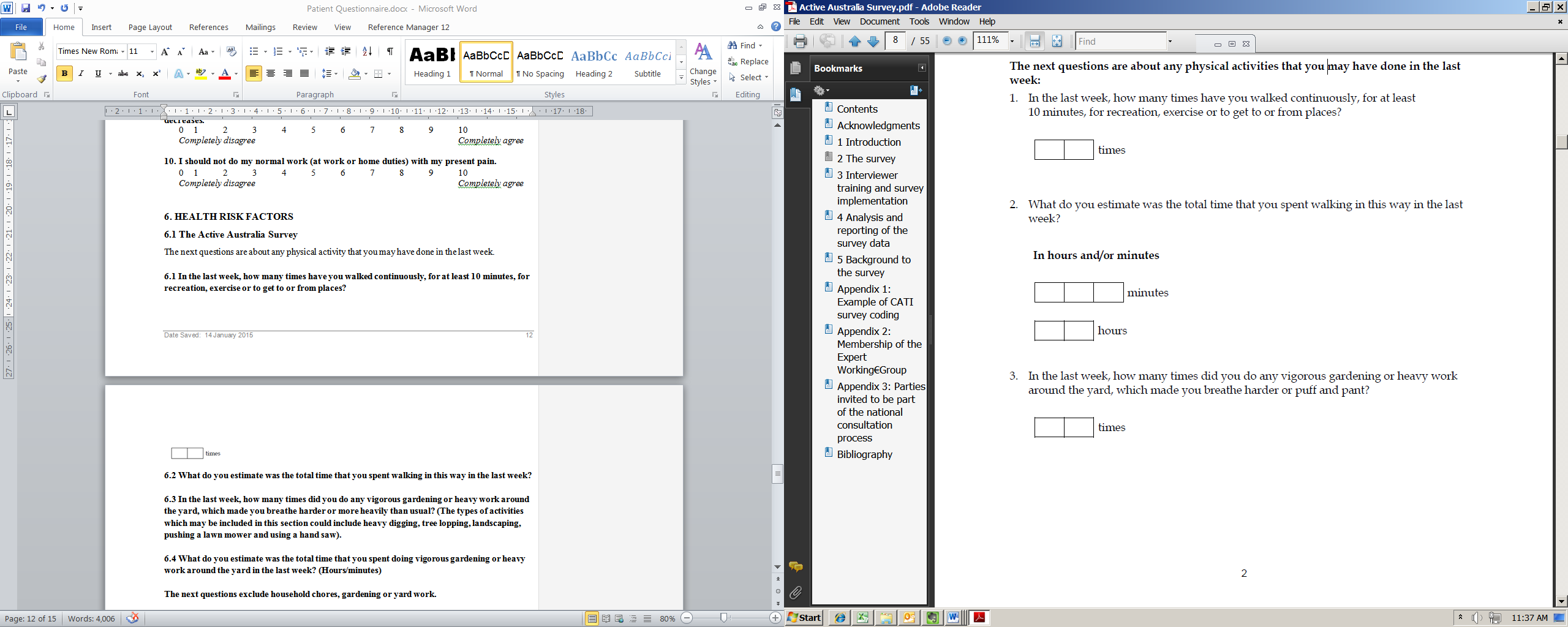


The next questions exclude household chores, gardening or yard work.

1. In the **last week**, how many times did you do any **vigorous physical activity** which made you breathe harder or more heavily than usual? (E.g. jogging, cycling, aerobics, competitive tennis, football, hockey, squash, hiking, weight lifting, boxing, basketball, netball, step aerobics)


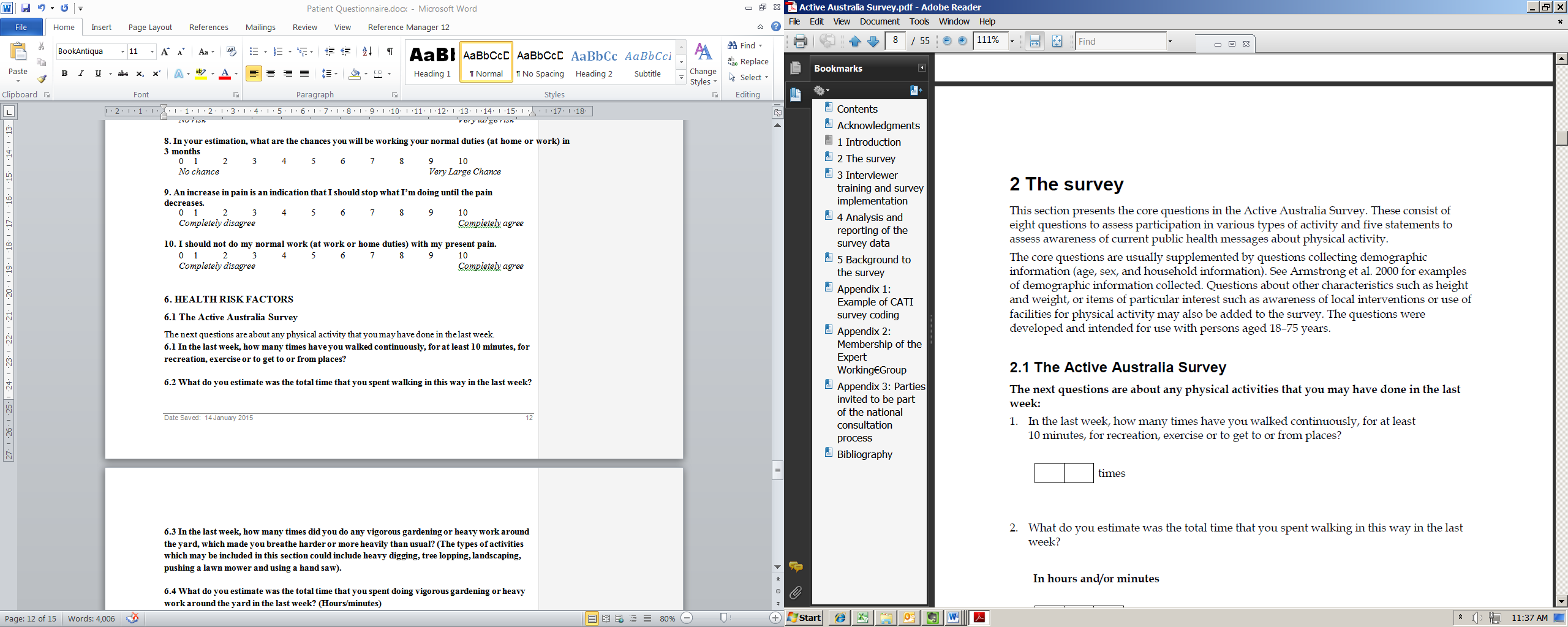


1. What do you estimate was the **total time** that you **spent doing this vigorous physical activity** in the last week?

In hours and/or minutes


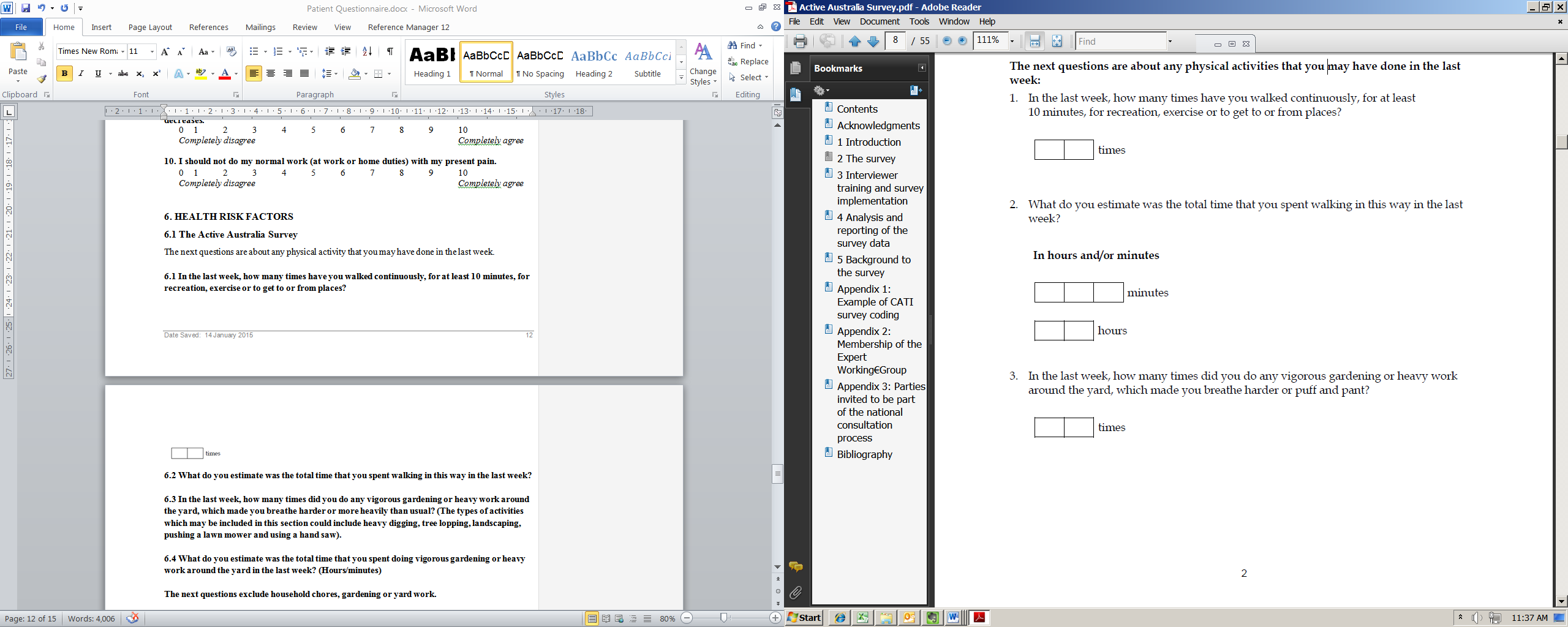


1. In the **last week**, how many times did you do any other more **moderate physical activities** that you have not already mentioned? (E.g. gentle swimming, social tennis, golf, dancing, table tennis, canoeing, cricket)


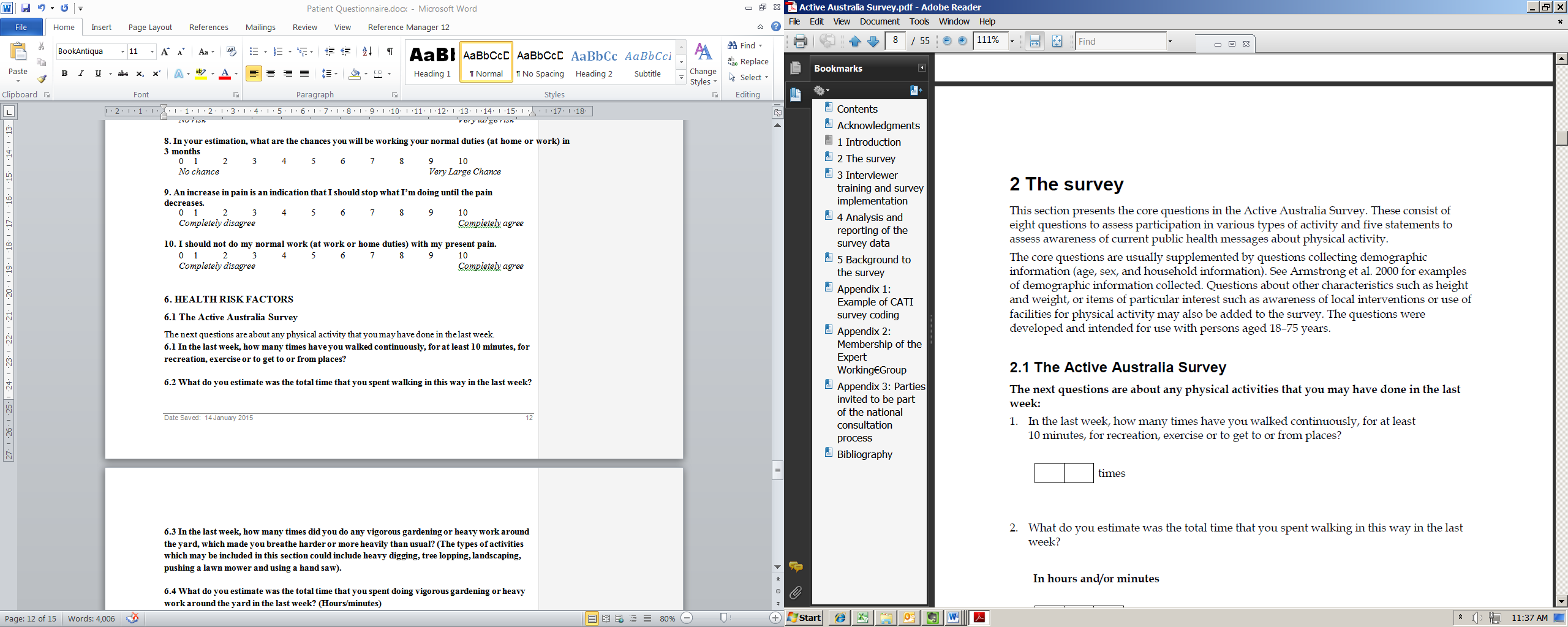


1. What do you estimate was the **total time** that you **spent doing these activities** in the last week?

In hours and/or minutes


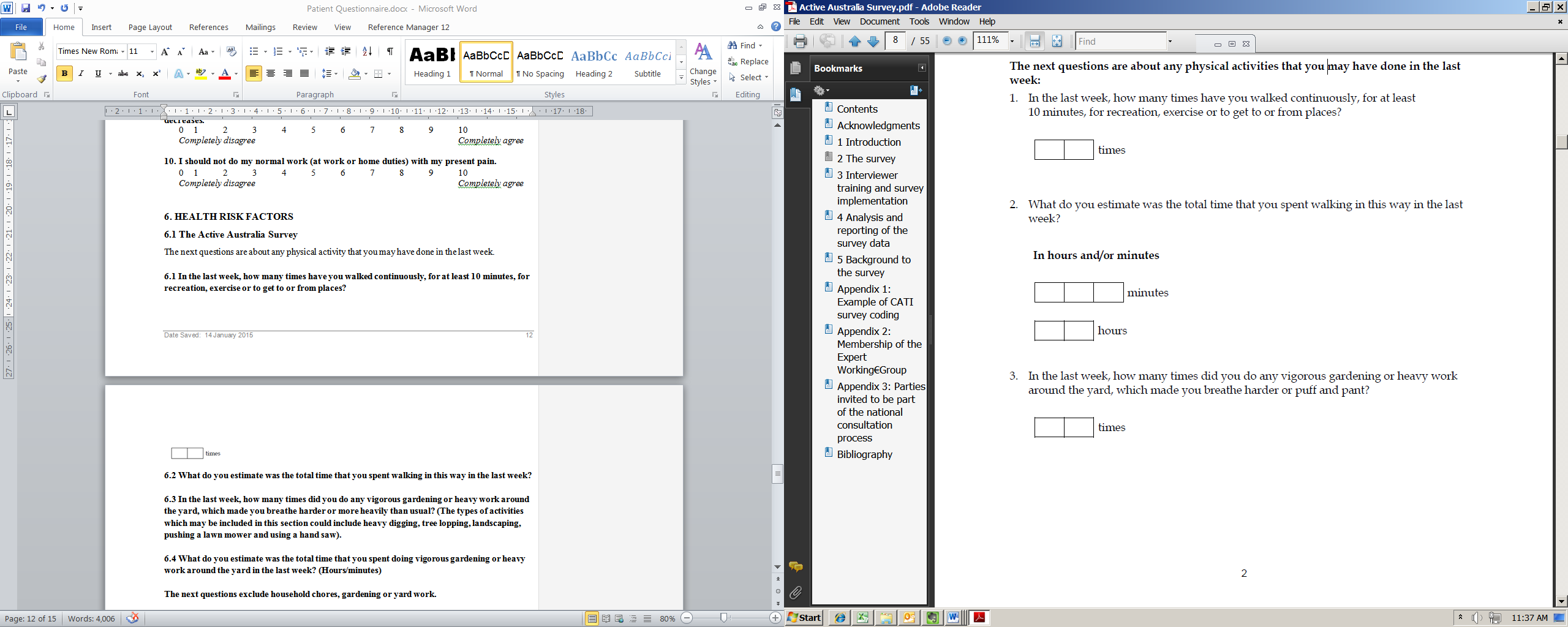


**Food Frequency Questionnaire**

The next questions ask you about your eating habits.

**Vegetables**

1. **How many serves of vegetables do you eat in a usual day?** *(*1 serve of vegetables is 75g or 1/2 cup cooked vegetables OR 75g or 1/2 cup cooked dried beans, peas or lentils OR 1 cup salad vegetables OR 1 potato*).*

🞎 0

🞎 1

🞎 2

🞎 3

🞎 4

🞎 5 or more

**Fruit**

1. **How many serves of fruit do you eat in a usual day?** *(*1 serve of fruit is: 1 medium apple, banana, orange, pear, OR 2 small pieces apricots, kiwi fruit, plums OR 1 cup diced pieces OR canned fruit OR 1/2 cup juice*).*

🞎 0

🞎 1

🞎 2 or more

**Meat**

1. **How often do you eat red meat, such as beef or lamb?** (Include all steaks, chops, roasts, mince, stir fries and casseroles. Do not include pork or chicken*).*

🞎 More than 1 time per day

🞎 Once per day

🞎 More than once a week

🞎 Once per week

🞎 More than once per month

🞎 Rarely or never

**Processed Foods**

1. **How often do you eat processed meat products such as sausages, frankfurts, devon, salami, meat pies, bacon or ham?**

🞎 More than 1 time per day

🞎 Once per day

🞎 More than once a week

🞎 Once per week

🞎 More than once per month

🞎 Rarely or never

1. **How often do you eat potato crisps or other salty snacks?** *(*such as Twisties or corn chips*)*

🞎 More than 1 time per day

🞎 Once per day

🞎 More than once per week

🞎 Once per week

🞎 More than once per month

🞎 Rarely or never

1. **How often do you have meals or snacks such as burgers, pizza, chicken or chips from places like McDonalds, Hungry Jacks, KFC, Red Rooster?**

🞎 More than 1 time per day

🞎 Once per day

🞎 More than once a week

🞎 Once per week

🞎 More than once per month

🞎 Rarely or never

1. **How often do you have snack foods such as sweet or savoury biscuits, cakes, doughnuts or muesli bars?**

🞎 More than 1 time per day

🞎 Once per day

🞎 More than once a week

🞎 Once per week

🞎 More than once per month

🞎 Rarely or never

1. **How often do you eat confectionary, such as lollies and chocolate?**

🞎 More than 1 time per day

🞎 Once per day

🞎 More than once a week

🞎 Once per week

🞎 More than once per month

🞎 Rarely or never

**Breads and cereals**

1. **How often do you usually eat bread?** *(*Include bread rolls, flat breads, crumpets, bagels, English or bread type muffins*)*

🞎 More than 1 time per day

🞎 Once per day

🞎 More than once a week

🞎 Once per week

🞎 More than once per month

🞎 Rarely or never

1. **How often do you eat pasta, rice, or noodles?**

🞎 More than 1 time per day

🞎 Once per day

🞎 More than once a week

🞎 Once per week

🞎 More than once per month

🞎 Rarely or never

**Drinks**

1. **How many cups of milk do you usually drink a day?** *(*1 cup = 250mL*)*

🞎 More than one cup per day

🞎 Around one cup per day

🞎 One cup per week

🞎 One cup more than once per week

🞎 One cup more than once per month

🞎 Rarely or never

1. **How many cups of soft drink, cordials or sports drink such as lemonade or Gatorade do you usually drink in a day?** *(*1 cup = 250mL, 1 can of soft drink = 1.5 cups, 1 x 500mL bottle of Gatorade = 2 cups*)*

🞎 More than one cup per day

🞎 Around one cup per day

🞎 One cup per week

🞎 One cup more than once per week

🞎 One cup more than once per month

🞎 Rarely or never

1. **How many cups of water do you usually drink a day?** *(*1 cup = 250mL*)*

🞎 More than one cup per day

🞎 Around one cup per day

🞎 One cup per week

🞎 One cup more than once per week

🞎 One cup more than once per month

🞎 Rarely or never

**Food Security**

1. **In the last 12 months were there any times that you ran out of food and couldn’t afford to buy more?**

🞎 Yes

🞎 No

🞎 Don’t know

**Alcohol Consumption**

For this section, alcohol drinks are measured in terms of a standard drink. A standard drink is equal to one middy of beer, one schooner of light beer, one small glass of wine or one pub-sized nip of spirits.

1. **How often do you have a drink containing alcohol?**

🞎 Never (finished questionnaire)

🞎 Monthly or less

🞎 2 to 4 times a month

🞎 2 to 3 times a week

🞎 4 or more times a week

1. **How many STANDARD DRINKS would you have on a typical drinking day?** (A standard drink is equal to one middy of beer, one schooner of light beer, one small glass of wine or one pub-sized nip of spirits)

🞎 1-2

🞎 3-4

🞎 5-6

🞎 7-9

🞎 10 or more

1. **How often would you have 4 OR MORE DRINKS on any occasion?**

🞎 Never

🞎 Less than monthly

🞎 Monthly

🞎 Weekly

🞎 Daily or almost daily

**Thank you! That is the end of the questionnaire**
